# Targeting BMAL1 reverses drug resistance of acute myeloid leukemia cells and promotes ferroptosis through HMGB1-GPX4 signaling pathway

**DOI:** 10.1007/s00432-024-05753-y

**Published:** 2024-05-04

**Authors:** Hong Zheng, Ting Wu, Zhi Lin, Dan Wang, Jing Zhang, Ting Zeng, Leping Liu, Jie Shen, Mingyi Zhao, Jia-Da Li, Minghua Yang

**Affiliations:** 1 Department of Pediatrics, The Xiangya Hospital, Central South University, Changsha, 410008 Hunan China; 2 Department of Pediatrics, The Third Xiangya Hospital, Central South University, Changsha, 410013 Hunan China; 3 Hunan Clinical Research Center of Pediatric Cancer, The Third Xiangya Hospital, Central South University, Changsha, 410013 Hunan China; 4 Center for Medical Genetics, School of Life Sciences, Central South University, Changsha, 410078 Hunan China; 5 MOE Key Lab of Rare Pediatric Diseases, The Third Xiangya Hospital, Central South University, Changsha, 410013 Hunan China

**Keywords:** Acute myeloid leukemias, Ferroptosis, BMAL1, Chemotherapy resistance

## Abstract

**Purpose:**

Acute myeloid leukemia (AML) is a refractory hematologic malignancy that poses a serious threat to human health. Exploring alternative therapeutic strategies capable of inducing alternative modes of cell death, such as ferroptosis, holds great promise as a viable and effective intervention.

**Methods:**

We analyzed online database data and collected clinical samples to verify the expression and function of BMAL1 in AML. We conducted experiments on AML cell proliferation, cell cycle, ferroptosis, and chemotherapy resistance by overexpressing/knocking down BMAL1 and using assays such as MDA detection and BODIPY 581/591 C11 staining. We validated the transcriptional regulation of HMGB1 by BMAL1 through ChIP assay, luciferase assay, RNA level detection, and western blotting. Finally, we confirmed the results of our cell experiments at the animal level.

**Results:**

BMAL1 up-regulation is an observed phenomenon in AML patients. Furthermore, there existed a strong correlation between elevated levels of BMAL1 expression and inferior prognosis in individuals with AML. We found that knocking down BMAL1 inhibited AML cell growth by blocking the cell cycle. Conversely, overexpressing BMAL1 promoted AML cell proliferation. Moreover, our research results revealed that BMAL1 inhibited ferroptosis in AML cells through BMAL1-HMGB1-GPX4 pathway. Finally, knocking down BMAL1 can enhance the efficacy of certain first-line cancer therapeutic drugs, including venetoclax, dasatinib, and sorafenib.

**Conclusion:**

Our research results suggest that BMAL1 plays a crucial regulatory role in AML cell proliferation, drug resistance, and ferroptosis. BMAL1 could be a potential important therapeutic target for AML.

**Supplementary Information:**

The online version contains supplementary material available at 10.1007/s00432-024-05753-y.

## Introduction

AML is caused by a series of acquired genetic abnormalities. Malignant transformation often occurs at the level of multipotent stem cells. Abnormal proliferation, clonal expansion, abnormal differentiation, and decreased apoptosis (programmed cell death) result in the malignant cells replacing normal blood cells (Peroni et al. 2023; Sauerer et al. 2023). The occurrence of AML has seen a notable rise in recent years. AML ranks first in the incidence of adult leukemia and accounts for approximately 25% of cases in pediatric acute leukemia (Khwaja et al. 2016). Currently, both have low cure rates, especially in adults, with a five-year survival rate of only 33% (Carter et al. 2020). Although the emergence of new treatment modalities in recent years, survival rates for AML have shown minimal improvement. The latest data indicates that there were 20,380 new cases of AML in 2023. From 1990 to 2020, the incidence rate of AML increased from 3.0 to 3.9 per 100,000 population (Vakiti and Mewawalla 2023). Between twenty-four to forty percent of children diagnosed with AML will encounter a relapse (Creutzig et al. 2012), and their long-term survival chances are approximately 30% (Kaspers 2012; Mustafa et al. 2019). Thus, there is a critical necessity to expedite the development of novel prognostic factors for monitoring the prognosis of AML patients and to explore innovative and effective therapeutic options.

BMAL1, as a core transcription factor in circadian rhythm, regulates downstream genes by binding to E-box elements within the promoter region. It can also function through heterodimerization with CLOCK protein (Miro et al. 2023). Recent studies have established the association of BMAL1 with tumor initiation, progression, and metastasis (Huang et al. 2023), as well as its correlation with chemotherapy resistance in tumor cells. There is considerable promise in the development of diverse therapeutic drugs targeting BMAL1 (Rasmussen et al. 2022). Ferroptosis is a form of programmed cell death that was first discovered in 2012 (Dixon et al. 2012). The essence of ferroptosis lies in the depletion of glutathione, the decrease in the activity of glutathione peroxidase 4 (GPX4), and the inability of lipid peroxides to be metabolized through the GPX4-catalyzed glutathione reductase reaction (Liu et al. 2023a, b). Subsequently, the oxidation of Fe2 + leads to the generation of reactive oxygen species (ROS), thereby promoting the occurrence of ferroptosis (Galy et al. 2023). According to current research findings, ferroptosis is associated with the pathological processes of various diseases, such as tumors (Zhu et al. 2023), diabetes (Zhang et al. 2023), neurodegenerative diseases (Wang et al. 2023a, b), ischemia–reperfusion injury (Xiang et al. 2023) and rheumatoid arthritis (Zhao et al. 2023). Ferroptosis is mainly regulated by the metabolism of system Xc-, GSH and GPX4 (Stockwell 2022). System Xc- is composed of a dimer of SLC3A2 and SLC7A11, which is embedded in the cell membrane surface. SLC7A11 is the main subunit of System Xc- and can transport cystine into cells for the synthesis of GSH. Therefore, inhibiting the expression of SLC7A11 can induce ferroptosis. P53 can downregulate the expression of SLC7A11 and inhibit the uptake of cystine (Li et al. 2020), leading to a decrease in GPX4, a reduction in cell antioxidant capacity, and an increase in sensitivity to ferroptosis. Current research indicates that GPX4 plays a pivotal role in regulating ferroptosis (Tang et al. 2021). GPX4 can reduce small molecule peroxides and certain lipid peroxides, protecting cells from oxidative damage. If GPX4 is deficient, phospholipid hydroperoxides can undergo catalytic reactions in the presence of excessive accumulated metals, such as iron, leading to cell death (Ursini and Maiorino 2020). Studies have found that downregulation of GPX4 expression in cells increases sensitivity to ferroptosis, while knocking down GPX4 induces ferroptosis (Stockwell et al. 2020). Conversely, upregulating GPX4 expression generates tolerance to ferroptosis. RSL3 directly binds to the GPX4 protein (Yang et al. 2014), rendering it inactive and inducing lipid ROS production, ultimately triggering ferroptosis. Macroautophagy (commonly referred to as autophagy) regulates ferroptosis by targeting and breaking down protein molecules that are essential for this cell death process. By selectively targeting and degrading ferroptosis repressors, including ferritin (Gao et al. 2016; Hou et al. 2016), BMAL1 (Yang et al. 2019), lipid droplets (Bai et al. 2019) and GPX4 (Wu et al. 2019), the promotion of ferroptosis occurrence is achieved. In our previous study, we uncovered the role of clockophagy in enhancing ferroptosis in HT1080 and Calu-1 cells. This mechanism revolves around the targeted breakdown of BMAL1 via the assistance of the cargo receptor SQSTM1/p62.This phenomenon takes place via the EGLN1-HIF1A pathway. It has been observed that when ferroptosis is induced by GPX4 inhibitors such as RSL3, there is a notable reduction in the protein stability of BMAL1. In contrast, our in vitro experiments demonstrated that SLC7A11 inhibitors, like erastin, had no impact on the stability of BMAL1. Studies have demonstrated that leukemia (Probst et al. 2017) and diffuse large B-cell lymphoma (Yang et al. 2014) exhibit sensitivity to ferroptosis. Targeting ferroptosis may hold promise as a novel treatment approach for cancer patients, particularly for those with AML who are resistant to traditional therapies (Hassannia et al. 2019; Liang et al. 2019). Nonetheless, the precise role that BMAL1 plays in modulating ferroptosis in AML is still not fully understood.

The BECN1 protein is an essential component in autophagy and serves as a key regulator in initiating the autophagic process (Dikic and Elazar 2018). HMGB1 is a non-histone chromosomasal binding protein that exists in the nucleus of eukaryotic cells. Research has shown its involvement in the pathological processes of various diseases, including tumors (Tang et al. 2023), chronic kidney disease (Liu et al. 2023a, b), autoimmune diseases (Ren et al. 2023), and cardiovascular diseases (Zheng et al. 2023). In the context of hematological malignancies, HMGB1 plays a pivotal role in modulating diverse biological processes, including cellular autophagy, differentiation, growth, immunity, and chemotherapy resistance (Yuan et al. 2020). In a study, it was demonstrated that HMGB1 exerted an upregulatory effect on TFRC expression via the MAPK pathway, consequently facilitating erastin-induced ferroptosis in leukemia cells (Ye et al. 2019). Based on our team's prior research, it is evident that a significant correlation exists between HMGB1 and autophagy. HMGB1, which interacts with BECN1 as a binding partner (Kang et al. 2010), can stimulate the formation of autophagosomes to induce autophagy (Tang et al. 2010). HMGB1-mediated autophagy, such as ferritinophagy, may contribute to increased iron accumulation during ferroptotic damage. Due to our prior research on BMAL1 and HMGB1, we were highly motivated to learn the functional relationship and specific roles of BMAL1 and HMGB1 in ferroptosis. However,the present understanding of this problem is still limited and required further investigation.

In this study, we primarily investigate the expression characteristics and functional roles of BMAL1 in AML, as well as its mechanism of regulating ferroptosis through HMGB1. Additionally, we also observe an association between BMAL1 and chemoresistance in AML. Our research has shown that BMAL1 is highly expressed in AML and is correlated with prognosis, as demonstrated through various experiments and analyses. BMAL1 impacts the growth rate of AML cells by influencing their cell cycle. We have also demonstrated that knockdown of BMAL1 can enhance the transcriptional expression of HMGB1. Elevated HMGB1 promotes autophagy-mediated degradation of GPX4, thereby facilitating the process of ferroptosis. Moreover, our study shows that the depletion of BMAL1 in AML cells leads to increased sensitivity to venetoclax, dasatinib, and sorafenib, both in laboratory cell cultures and animal models. This study emphasizes that targeting BMAL1 can enhance sensitivity of AML cells to ferroptosis inducer RSL3, as well as the tumor treatment drugs venetoclax, dasatinib, and sorafenib. Molecular inhibitors targeting BMAL1 could be developed for clinical AML treatment to improve therapeutic efficacy in the future.

## Materials and methods

### Cell line and culture

HL60 and MOLM13 cell lines were cultured using RPMI 1640 medium (Gibco, USA), while the 293T cell line was cultured using DMEM medium. The culture media were supplemented with 10% fetal bovine serum (VivaCell, China) and 1% penicillin–streptomycin (Abiowell, China) to provide optimal conditions for cell growth and viability. The prepared medium was stored at 4 °C to maintain its freshness and prevent contamination.The cells were cultivated in a sterile culture dish under controlled conditions. The cultivation environment included a temperature of 37 degrees Celsius and a carbon dioxide concentration of 5%, which mimicked the physiological conditions suitable for cell growth and proliferation. To ensure the integrity and purity of the cell cultures, all procedures were carried out in an aseptic environment within a dedicated cell culture facility. The cells were handled with utmost care to minimize any potential contamination. Regular checks for mycoplasma contamination were performed, and the results were consistently negative, affirming the high-quality of the cell lines used. During the experimental process, the appropriate size of culture dishes was selected for plating the cells. The cell density in each dish was carefully controlled by counting the cells using a specialized cell counting board, allowing for consistent and reproducible results across experiments. For drug treatments, the drugs were prepared using dimethyl sulfoxide (DMSO). To ensure minimal impact on the cells, the concentration of DMSO in the experiments was maintained below 0.01%. If the concentration exceeded this threshold, the control group received the corresponding volume of DMSO to maintain consistency in the experimental conditions. In cases where drugs were prepared using other solvents, the control group received the equivalent volume of the solvent to ensure accuracy and reliability in the experimental design. After plating, the culture environment was meticulously maintained to mirror the standard cell culture conditions. This ensured that the cells experienced a consistent and optimal growth environment, allowing for reliable and meaningful observations throughout the course of the experiments.

### Animal models

Male nude mice aged 6–8 weeks were selected for our study. These mice were procured from Hunan SJA Laboratory Animal Co., Ltd(China), a reputable supplier known for providing high-quality laboratory animals. To maintain a controlled environment conducive to the mice's well-being, they were housed in a specific pathogen-free facility with appropriate temperature and humidity levels. A 12-h light–dark cycle was implemented to mimic natural day-night conditions. To ensure the hygiene and safety of the mice, sterilized feed and water were provided throughout the experiment. The growth density of the mice adhered to established hygiene standards, and their growth status was closely monitored on a daily basis. This allowed us to promptly identify any signs of illness or distress and take appropriate action, ensuring the mice remained healthy and robust. During the tumor modeling process, confounding factors such as gender, age, and weight were carefully considered and balanced. Random assignment of mice into experimental groups was performed to minimize bias and ensure the reliability of our results. Subcutaneous tumor induction in mice was achieved using HL60 cells at a density of (2 × 106)/100 μl, suspended in phosphate-buffered saline. The injection site on the back of the mice was chosen to avoid hindering their movement or causing discomfort. After approximately one week, we confirmed the successful tumor formation in the mice, meeting the requirements of a tumor volume between 90-110mm3. Throughout the entire experimental process, we strictly adhered to ethical standards, ensuring that the tumor volume did not exceed 2000mm3, thereby preventing unnecessary harm to the animals. The drugs used in the animal experiments were sourced from Selleck, a reputable supplier known for providing high-quality compounds for research purposes. The preparation and administration of the drugs followed the specific standards provided by each drug's manufacturer, ensuring accuracy and consistency. For intraperitoneal injections, a stock solution was prepared at a concentration of 125 mg/ml using a mixture of 5% DMSO, 40% PEG300, 5% Tween80, and 50% ddH2O. The working solution for injection had a concentration of 6.25 mg/ml. In the case of oral administration, the solvent was prepared fresh and immediately before use, with the components (4% DMSO, 30% PEG300, 5% Tween80, and ddH2O) added sequentially to the product from left to right. To monitor the mice's health and response to the experimental treatments, their body weight was periodically measured and recorded as per the requirements of the study. This allowed us to evaluate any potential effects of the drugs on the mice's overall well-being. At the conclusion of the experiment, the mice were euthanized in a humane manner, following approved protocols. Organs and tissues relevant to the study were collected for further analysis and experimentation, contributing valuable insights to our research endeavor.

### Immunoblotting

The cells were collected using a centrifugation speed of 2000r/min for 3 min to ensure the efficient collection of all cells. To remove any residual contaminants, the cells were washed once with pre-chilled PBS. Following this, the cells were centrifuged again to collect them, and then lysed using a prepared RIPA cell lysis buffer. The RIPA lysis buffer was supplemented with 1X proteinase inhibitor and phosphatase inhibitor to maintain the integrity of the proteins during the lysis process.To prevent protein degradation, the entire process of cell lysis was conducted on ice, maintaining a low temperature throughout. After cell lysis, the cells were sonicated to further disrupt the cellular structures and release the proteins of interest. The sonication conditions were carefully set as follows: 30 watts of power, 3 s of sonication followed by a 10-s rest period, repeated 5 times. This ensured effective disruption without excessive heat generation. To quantify the protein concentration in the cell lysates, the BCA assay kit (Thermo Fisher Scientific) was used. This assay allowed for accurate measurement of protein content based on the colorimetric detection of the reaction between proteins and the BCA reagent. Following the quantification, the cell lysates were mixed with the loading buffer and boiled for 10 min. This step denatured the proteins and prepared them for subsequent gel electrophoresis. For gel electrophoresis, each protein sample, approximately 30 µg in mass, was loaded onto an SDS-PAGE gel. The electrophoresis conditions were set initially at 70 V for 30 min to allow for efficient sample migration through the stacking gel. Subsequently, the voltage was adjusted to 120 V, with the time adjusted based on the desired resolution and separation of the target protein bands. After electrophoresis, the proteins were transferred from the SDS-PAGE gel to a PVDF membrane using a wet transfer system. The transfer conditions involved a constant current of 290mA for 90 min, ensuring efficient and complete transfer of the proteins to the membrane. To prevent non-specific binding and enhance the specificity of antibody binding, the PVDF membrane was blocked at 4 degrees Celsius for 2 h. The blocking buffer consisted of 5% milk in TBST, which effectively blocked any remaining unoccupied binding sites on the membrane. Following the blocking step, the primary antibody, specific to the protein of interest, was incubated with the membrane overnight at 4 degrees Celsius. The antibody dilutions were prepared according to the recommendations provided by the antibody manufacturers, ensuring optimal antibody-antigen interaction and detection. The choice of primary antibodies relied on the specific target proteins being investigated in the experiment. These antibodies were carefully selected based on their known specificity and affinity towards the proteins of interest. The antibodies used were as follows:

ACTB: AC026, 1:5000 dilution, Abclonal.

BMAL1: 14,040, 1:500 dilution, Cell Signaling Technology.

GPX4: 125,066, 1:1000 dilution, Abcam.

P62: A19700, 1:1000 dilution, Abclonal.

HMGB1:3935, 1:3000, Cell Signaling Technology.

LC3A/B: 4108, 1:1000 dilution, Cell Signaling Technology.

The secondary antibody, diluted at a ratio of 1:10,000, was carefully incubated with the membrane for 2 h at 4 degrees Celsius to ensure specific binding to the primary antibody-antigen complex. Following this incubation, the membrane was subjected to a thorough washing process using 0.1% TBST, with each wash lasting 7 min and a total of 3 washes being performed. This rigorous washing step effectively removed any unbound or non-specifically bound antibodies, minimizing background noise and ensuring the specificity of signal detection.To further ensure the removal of residual washing buffer, the membrane was then washed once with TBS, providing a clean and neutral environment for subsequent chemiluminescent detection.For the final step of signal detection, either the highly sensitive SuperBright Subpico ECL (SUDGEN, catalog no. 31060) or the Super-sensitive ECL chemiluminescent substrate (Biosharp, catalog no. BL520B) was employed. These substrates offered exceptional sensitivity and signal strength, allowing for the precise visualization and detection of the target proteins on the membrane. The choice between the two substrates depended on the specific experimental requirements and the desired balance between sensitivity and signal duration.

### Quantitative PCR analysis for gene expression

RNA extraction was conducted using proper personal protective equipment, including gloves, masks, and protective clothing, to minimize RNA degradation. Initially, cells were centrifuged at 1000 rpm for 3 min to form a cell pellet, after which the culture medium was removed. Subsequently, 1 ml of TRIzol was added to each 5–10 × 10^6 cells and thoroughly pipetted at room temperature for 10 min to lyse the cells. Following this, 200 μl of chloroform was added to each 1 ml of TRIzol, and the mixture was vigorously shaken by hand for 15 s before being incubated at room temperature for 2–3 min. The resulting mixture was then centrifuged at 12,000 g for 15 min at 4 degrees Celsius, and the aqueous phase was carefully transferred to a new EP tube. Next, 600 μl of isopropanol was added, and the mixture was inverted several times before being placed at -20 degrees Celsius for 30 min. After centrifugation at 4 degrees Celsius for 10 min at 12,000 rpm, the supernatant was discarded. Subsequently, 1 ml of 75% ethanol was used to wash the pellet twice, with the mixture being centrifuged at 4 degrees Celsius for 5 min at 7500 g each time. After ethanol washing, the supernatant was removed, and the pellet was air-dried at room temperature for 5–10 min. Finally, the RNA was dissolved in DEPC-treated water and stored at -80 degrees Celsius. For RNA integrity assessment, a 1.2% agarose gel was prepared, and the RNA samples were mixed with RNA loading buffer and loaded into the wells for electrophoresis. UV imaging revealed three distinct bands from top to bottom: 28s RNA, 18s RNA, and 5s RNA. The 28s RNA band should exhibit twice the intensity of the 18s RNA band and should not display any smearing, indicating high-quality RNA. Subsequently, RNA reverse transcription was performed using the Takara Reverse Transcription Kit (RR037A) according to the provided manual. Real-time quantitative PCR was then carried out using ChamQ Blue Universal SYBR qPCR Master Mix (Q312-02, Vazyme) in a 10 μl reaction system, incorporating 5 μl of qPCR Master Mix, 0.4 μl of each forward and reverse primer, and the remaining volume filled with DNA and ddH2O. The CFX96 Real-Time System (Bio-Rad) was utilized for the qPCR analysis, with actin serving as the internal reference for data analysis. To ensure experimental accuracy, each experiment was independently repeated three times.

### Cell proliferation assays

Cell viability experiments were performed using the highly sensitive CCK-8 assay kit (GK3607-500T, DING GUO PROSPEROUS) for accurate detection and analysis. To establish the experimental setup, a 96-well plate was chosen as the plating platform, with each well initially seeded with a cell density of 5 × 10^4 cells. Prior to each experiment, precise cell counting was conducted using a specialized cell counting chamber, allowing for the calculation of the required volume of cell suspension based on the desired cell density.Once the cell suspension was prepared, it was thoroughly mixed and dispensed into individual labeled EP tubes. Simultaneously, the drugs or compounds to be tested were appropriately prepared and added to their respective labeled tubes. To ensure comprehensive exposure of the cells to the drugs, a meticulous mixing step was carried out, ensuring proper distribution and interaction between the cells and the test substances. Subsequently, 100 μl of the well-mixed cell suspension was carefully pipetted into each well of the 96-well plate, ensuring uniform distribution across all wells. The plated cells were then placed in a precisely controlled incubator, providing an optimal cultivation environment conducive to cell growth and drug treatment. Following the designated period of drug treatment, 10 μl of the CCK-8 detection reagent was added to each well with utmost care to prevent the formation of bubbles. This reagent is specifically designed to assess cell viability, providing reliable and quantitative results. After an incubation period of 1–2 h to allow for proper reaction, the absorbance of the samples at 450nm was measured using a high-performance microplate reader.

### MDA assay

To ensure the accurate and reliable detection of malondialdehyde (MDA), an important marker of oxidative stress, the MDA assay kit from Beyotime (S0131M) was utilized. First, cells were collected by centrifugation at 1000 rpm for 3 min and washed with PBS to remove any extraneous substances. For every 1 × 10^6 cells, 100 μl of ice-cold lysis buffer was added, and the cells were gently lysed on ice for 15–20 min. The lysate was then centrifuged at 12,000 g for 10 min, and 100 μl of the supernatant was collected for subsequent measurements.To establish a standard curve, the blank control and standard samples at concentrations of 0, 3.125, 6.25, 12.5, 25, and 50 μM were prepared. In the control, standard, and test samples, 200 μl of the working solution was added. After thorough mixing, the samples were heated at 100 °C for 15 min to promote the reaction between MDA and the working solution. The samples were then allowed to cool in a water bath and centrifuged at 1000 g for 10 min at room temperature. Next, 200 μl of the supernatant was transferred to a 96-well plate, and absorbance was measured at 532 nm using a microplate reader.To account for variations in protein concentration across different samples, the protein content of the test samples was determined using the BCA method. The MDA content in the samples was then calculated as units per unit weight of protein. Standardization was performed using the control group to ensure consistency and accuracy across all experiments.

### Lipid peroxidation probe -BDP 581/591 C11-

We used Lipid Peroxidation Probe -BDP 581/591 C11 for lipid peroxidation detection(Dojindo Laboratories, L267). The protocol we used was in accordance with the manufacturer's instructions, and involved following the specific steps outlined below: HL60 and MOLM13 cells were plated in a 12-well plate at a density of 6*10^5 cells/ml, with RSL3 at a concentration of 0.5 μM. After incubating in a cell culture incubator for 24 h, the culture medium was discarded. The cells were then washed twice with HBSS by centrifuging at 2000 rpm for 3 min to collect them. After removing the HBSS, probe working solution was added and the cells were incubated in the cell culture incubator for 30 min. The working solution was subsequently removed, and the cells were rinsed twice with HBSS before adding 500 µl of HBSS per well. Finally, the cells were observed and photographed using a fluorescence microscope.

### Cell cycle analysis

To ensure accurate and precise analysis of cell cycle distribution, the following optimized protocol was implemented. First, the processed cells were collected, and to prepare a cell suspension, 300 μl of pre-chilled PBS was added. Subsequently, 900 μl of pre-chilled absolute ethanol was added dropwise while continuously shaking, resulting in a final ethanol concentration of 75%. The samples were then placed in a 4-degree environment for 24 h to fix the cells.To proceed with cell cycle analysis, the fixed cell suspension was centrifuged at 1000 rpm for 5 min, and the supernatant was carefully discarded. Next, 1 ml of pre-chilled PBS was added to resuspend the cells, which were then subjected to another centrifugation step at 1000 rpm for 5 min to collect the cells. This washing step was repeated twice to remove any remaining ethanol. Following the removal of ethanol, 200 μl of propidium iodide (PI) working solution was added to the cell suspension, and the cells were stained for 30 min in the dark at 4 °C. Subsequently, the cells were centrifuged at 1000 rpm for 5 min to obtain a cell pellet, which was washed once with PBS to remove excess PI staining. For flow cytometric analysis, the cells were transferred to a flow cytometry tube and analyzed using a flow cytometer. The PI fluorescence was excited by a 488 nm argon ion laser and detected through a 630 nm bandpass filter. To ensure reliable data acquisition, a total of 10,000 cells were collected based on forward scatter (FSC) and side scatter (SSC) scatter plots. Gate technology was employed to exclude adherent cells and debris, ensuring that only viable cells were included in the analysis.

### Transmission electron microscope (TEM)

The experiment involved several pivotal stages, including fixation, dehydration, infiltration, embedding, sectioning, staining, and imaging. In the fixation phase, cells were initially exposed to a 2.5% glutaraldehyde solution for a duration of 6–12 h. Subsequently, they were transferred to a PBS buffer solution for 1–6 h after removing the fixative, followed by fixation using a 1% osmium tetroxide solution for 1–2 h. Dehydration involved a sequential series of exposure, starting with 30% ethanol for 10 min, followed by 50% ethanol for another 10 min. The cells underwent an immersion in a 70% uranyl acetate solution for either 3 h or overnight. This was followed by exposure to 80% ethanol for 10 min, 95% ethanol for 15 min, and two rounds of 100% ethanol for 50 min each. Finally, the cells received a 30-min treatment with epoxypropane. In the infiltration phase, the cells were immersed in a mixture of epoxy resin and epoxypropane in a 1:1 ratio for a duration of 1–2 h. They were then soaked in pure epoxy resin for an additional 2–3 h. For embedding, the cells were fully immersed in pure epoxy resin and incubated in a 40 °C oven for 12 h. This was followed by further incubation in a 60 °C oven for 48 h. Sectioning involved cutting ultra-thin slices from the embedded block, and these slices were retrieved using copper grids. The staining process consisted of electron staining utilizing lead and uranium, while imaging was accomplished by observing the samples through a JEOL JEM1400 transmission electron microscope and capturing images using a Morada G3 digital camera.

### Lentivirus infection

To optimize gene overexpression and knockdown experiments, we obtained short hairpin RNAi molecules targeting BMAL1 and HMGB1, as well as the necessary overexpression plasmids, from Genechem CO.LTD (Shanghai, China). Lentiviruses were then prepared by transfecting 293T packaging cells in 10 cm dishes using Lipo8000 (No. C0533, Beyotime) as the transfection reagent. To ensure optimal virus packaging, the following steps were taken: On the first day, 293T cells were seeded in a 10 cm dish with a density controlled at around 70–80% for transfection the next day. On the second day, the constructed plasmids and virus packaging plasmids were co-transfected into 293T cells at a certain ratio. After 48 h of transfection, the supernatant virus liquid was collected via centrifugation at 1500 rpm for 5 min and then filtered using a 0.45 μm filter. The resulting virus liquid can be used for subsequent experimental transfections and is stored at -80 degrees Celsius for long-term preservation.After preparing the lentiviruses, they were introduced into HL60 and MOLM13 cells for transduction. We evaluated multiple hairpin constructs to select those with the highest knockdown efficiency. To ensure optimal infection efficiency based on the different characteristics of virus-infected cells, appropriate volumes of the virus were used to infect the cells. After 24 h of viral infection, the cells were replenished with fresh cell culture medium, and corresponding antibiotics were used for selection for three days based on the resistance carried by the transfected plasmid. The efficiency of plasmid transfection was determined through RNA or protein level detection.

### Chromatin immunoprecipitation (ChIP)

The ChIP assay was conducted following the protocol outlined in the ChIP Kit (No. ab500, Abcam). The steps of the experiment could be divided into cell fixation and collection, cell lysis, immunoprecipitation, and DNA purification. The steps for the cell fixation experiment were as follows: firstly, 288 μL of 37% formaldehyde (final concentration of 1%) was slowly added along the wall of a 10 cm cell culture dish containing 10 mL of medium, and incubated at room temperature on a shaker for 10 min. Then, 1 mL of 1.25 M glycine was added to neutralize the unreacted formaldehyde, and the mixture was incubated at room temperature on a shaker for 5 min. After washing twice with pre-chilled PBS, the cells were collected using a cell scraper and transferred to a 1.5 mL enzyme-free centrifuge tube containing 1 mL of PBS. The tube was then centrifuged at 4 °C, 1300g for 5 min using a refrigerated centrifuge, and the supernatant was discarded. The cell sonication conditions were set to obtain desired DNA fragments ranging from 200 to 1000 bp in size. Antibodies against BMAL1 (No.14020s, Cell Signaling Technology), IgG control (No.2729, Cell Signaling Technology), and Flag (F4049, Sigma) were used along with ChIP-Grade Protein G Magnetic Beads for the immunoprecipitation step. DNA purification was performed using DNA purification magnetic beads.The resulting ChIP-enriched chromatin was subjected to qPCR analysis. The final analysis results were expressed as a ratio relative to the input DNA. The specific primers used for PCR amplification are provided below:F-P1: GCTGACGAAAGAGACCTGCT; R-P1: CTTCGGAAGCCCTTCCCTC. F-P2: CTCCTTTCCTCCCTCCCAGA; R-P2: GGACAGATCGGCTGTTGACT. F-P3: GCAGTACCTTCCAGTGGGATT; R-P3: AAGCTTCCTCCCTTTAAATCATGT.

### Luciferase assay

On the first day, 293T cells were seeded into a 24-well plate, with a seeding density of 70–80% confluency targeted for transfection on the following day. On the second day, plasmid transfection was performed with a total of 200 ng of plasmid DNA per well. This included 1 ng of Renilla plasmid, and the transfection ratio of luciferase plasmid to the target plasmid was adjusted according to experimental requirements, with the remaining plasmid volume being made up with empty vector as needed. After transfection, the cells were treated with RSL3 and incubated for 24 h. Fluorescence quantification was performed 48 h after transfection completion. The cells were collected, and a Dual-Luciferase reporter system (Promega, USA) was used for fluorescence detection. The 24-well plate was washed once with PBS. After adding 100 uL of PLB dilution buffer to each well, it was gently shaken on a shaker at low speed for at least 30 min. The lysate was then aspirated into an EP tube and centrifuged at 15,000 rpm, 4 °C, for 30–60 s. One EP tube was taken, and 20 uL of LAR II and 4 uL of cell lysate supernatant were added. After thorough mixing, the first luminescence reading (experimental value) was measured. Subsequently, 20 uL of S&G mixing solution was added to the EP tube and mixed thoroughly, followed by measurement of the second luminescence reading (internal reference value). Each treatment had three replicate wells, and three independent experiments were performed.

### Statistical analysis

A p-value less than 0.05 was considered to indicate significant differences. Student unpaired t-test were used for comparison between two groups, and, one-way or two-way ANOVA was used for comparison among multiple groups. Before conducting any significance tests, all data underwent tests for data applicability. Data analysis was performed using GraphPad Prism software.

## Results

### BMAL1 functions as a positive modulator in AML cells

**Fig. 1:**
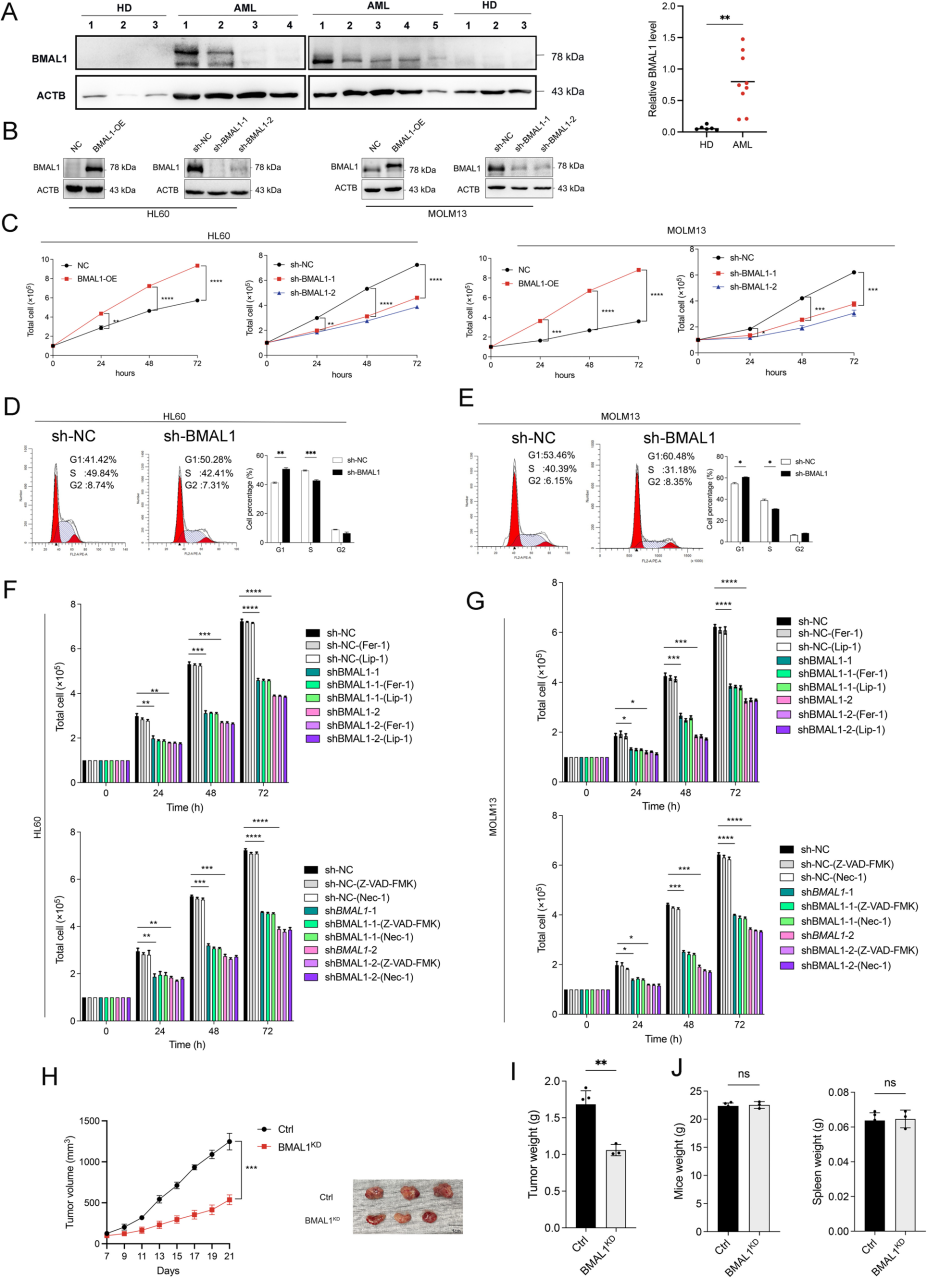
BMAL1 functions as a positive modulator in AML cells. **A** Western blots detection of the level of BMAL1 expression in AML patients and peripheral mononuclear cells of healthy donors (HD). ACTB was used as an internal control (left). The density of the protein band was quantified and plotted on graph (right). **B** The lentiviral-mediated overexpression and knockdown of BMAL1 in HL60 and MOLM13 cell lines following transfection. **C** Cell growth was measured by manual cell counting. The number of both control BMAL1-overexpressing and BMAL1-depleted HL60 and MOLM13 cells were counted by typhan blue exclusion method at the indicated time points. **D–E** Cell cycle in HL60 and MOLM13 cells with BMAL1 knockdown. **F–G** Cell growth was measured by manual cell counting. The number of both control and BMAL1-depleted HL60 (**H**) and MOLM13 (**I**) cells were counted by trypan blue exclusion method at the indicated time points following treatment with ferrostatin-1 (0.5 μM), liproxstatin-1 (0.5 μM), Z-VAD-FMK (20 μM),or necrostatin-1 (30 μM). **H–I** The sizes and weights of xenograft tumors from nude mice (three mice per group) were compared between BMAL1 knockdown and control groups. **J** The weight of each mouse and spleen were recorded at the end of the observation period. Data represents the mean ± SD from three independent experiments; n.s. (no significance).Statistical significance in (**A**), **I–J** was calculated by two-tailed unpaired t test, by two-way ANOVA with Dunnett’s multiple comparison test for (**C**–**H**). *p < 0.05; **p < 0.01; ***p < 0.001. ****p < 0.0001

We conducted a bioinformatics analysis utilizing the Cancer Genome Atlas (TCGA) dataset to investigate the association between BMAL1 expression and AML. Our analysis revealed notable variations in the expression of BMAL1 across 14 different types of tumors, as depicted in Supplementary Fig. 1A. In particular, among the 14 different types of tumors, BMAL1 was found to be highly expressed only in AML. This discovery indicates the potential for a distinctive functional role of BMAL1 in AML. Afterwards, we carried out an in-depth analysis to explore the potential correlation between BMAL1 expression levels and patient survival outcomes.The data presented in Supplementary Fig. 1B clearly illustrates that patients with elevated BMAL1 expression had considerably shorter overall survival durations compared to their counterparts with low BMAL1 expression. This highlights the potential of BMAL1 expression as a prognostic marker for AML, indicating its significance in predicting patient outcomes. In order to reinforce our findings, we collected bone marrow cells from newly diagnosed AML patients (with more than 90% blast cells in morphology assessment) and mononuclear cells from peripheral blood of healthy donors. Our results indicated a notable upregulation of BMAL1 expression in AML patients. (Fig. 1A), although the levels of BMAL1 in AML cells are not significantly higher compared to other cell types (Supplementary Fig. 1C). We have provided the clinical characteristics of the AML patients involved in our study in Supplementary Table 1. Our analysis revealed an intriguing finding: the expression of BMAL1 can be utilized as a prognostic indicator for survival in AML patients.

To elucidate the functional role of BMAL1 in AML, a series of functional experiments were performed using AML cell lines HL60 and MOLM13. Our results indicated that overexpression of BMAL1 significantly enhanced cell proliferation in both HL60 and MOLM13 cells (Fig. 1B, C). Conversely, depletion of BMAL1 by shRNAs in HL60 and MOLM13 cells reduced cell proliferation (Fig. 1B, C). In order to elucidate the causes of the attenuated growth of AML cells following BMAL1 knockdown, we conducted distinct experiments including cell cycle flow cytometry analysis and the application of cell death inhibitors such as apoptosis inhibitors, necroptosis inhibitors, and ferroptosis inhibitors. Our findings revealed a significant induction of G1 to S cell cycle arrest upon depletion of BMAL1 in HL60 and MOLM13 cells (Fig. 1D, E). But we observed that the inhibitors targeting apoptosis, necroptosis, and ferroptosis did not reverse the proliferation of AML cells affected by BMAL1 depletion (Fig. 1F, G, Supplementary Fig. 1D).

The xenograft model of leukemia cells is commonly used to study acute leukemia in *vivo* (Zhou et al. 2021; Wang et al. 2022; Zhang and Su 2022). In order to further elucidate the role of BMAL1 in AML progression, we injected stable BMAL1-depleted HL60 cells or empty vector-transfected (control) cells into BALB/c mice. In concurrence with our in vitro experimental results, the depletion of BMAL1 exhibited a pronounced deceleration in tumor growth, characterized by notable reductions in tumor volume and weight (Fig. 1H, I). Of note, our study did not reveal any significant alterations in the body weight or spleen weight (Fig. 1J) of the host mice following tumor inoculation, suggesting the absence of any adverse effects on the health of the animals. This observation further supports the notion that BMAL1 functions as a key positive regulator of AML tumorigenesis.

### BMAL1 as an inhibitor of ferroptosis in AML cells

As demonstrated in our previous investigation, clockphagy refers to the specific degradation of BMAL1 and is mediated by the cargo receptor SQSTM1/p62 (Yang et al. 2019). Our observations revealed that BMAL1 functioned as an inhibitor of ferroptosis and underwent degradation when exposed to the ferroptosis inducer RSL3 (GPX4 inhibition), rather than Erastin (SLC7A11 inhibition). This degradation process subsequently promoted the progression of ferroptosis. The expression of BMAL1 was upregulated in AML patients when compared to the control group. Based on these findings, it is postulated that BMAL1 could have a crucial involvement in the process of ferroptosis in AML.

**Fig. 2:**
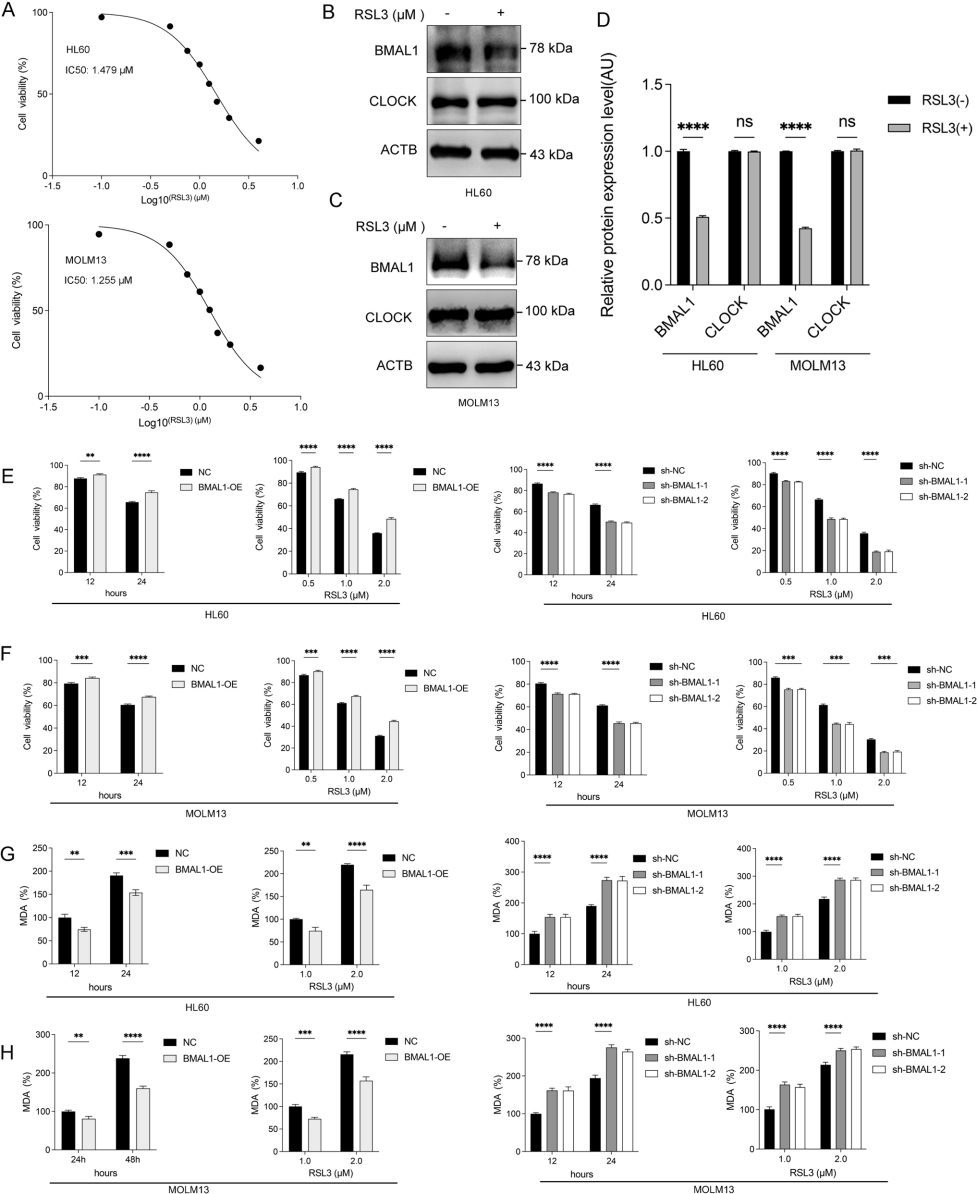
BMAL1 as an inhibitor of ferroptosis in AML cells. **A** Cell viability of HL60 and MOLM13 cells following treatment with RSL3 at the indicated concentration for 24h. **B–C** Western blots detection of the level of BMAL1 expression in HL60 and MOLM13 cells following treatment with RSL3(1.25 μM) for 24h. **D** The relative level of protein quantification for (B-C). **E** Viability of both controls, BMAL1-overexpressing and BMAL1-depleted HL60 cells following treatment with RSL3 at the indicated time points (RSL3: 1.0 μM, left) and at the indicated concentration (time: 24 h, right). **F** Viability of both controls, BMAL1-overexpressing and BMAL1-depleted MOLM13 cells following treatment with RSL3 at the indicated time points (RSL3: 1.0 μM, left) and at the indicated concentration (time: 24 h, right). **G** Analysis of MDA levels in both controls, BMAL1-overexpressing and BMAL1-depleted HL60 cells following treatment with RSL3 at the indicated time points (RSL3: 1.0 μM) and at the indicated concentration (time: 24 h). **H** Analysis of MDA levels in both controls, BMAL1-overexpressing and BMAL1-depleted MOLM13 cells following treatment with RSL3 at the indicated time points (RSL3: 1.0 μM) and at the indicated concentration (time: 24 h, 48h). Data represents the mean ± SD from three independent experiments; n.s. (no significance), Statistical significance in (**D**–**H**) was calculated by two-way ANOVA with Dunnett’s multiple comparisons test. *p < 0.05; **p < 0.01; ***p < 0.001. ****p < 0.0001

Initially, we utilized the CCK8 assay to assess the sensitivity of HL60 and MOLM13 cells to RSL3, and the dose–response curve is illustrated (Fig. 2A). The IC50 values for HL60 and MOLM13 cells were 1.479 μM and 1.255 μM, respectively. In order to explore the involvement of BMAL1 in AML ferroptosis, we evaluated changes in protein and mRNA expression levels of BMAL1. The Western blot results showed a decrease in BMAL1 protein levels under the influence of RSL3, with CLOCK protein serving as a positive control (Fig. 2B–D). The qPCR results indicated that there were no significant changes in the mRNA levels of BMAL1 when RSL3 was used alone or in combination with the ferroptosis inhibitor ferrostatin-1 or liproxstatin-1 (Supplementary Fig. 2A, B). However, the mRNA levels of *PER1* and *CRY1*, which are transcriptionally regulated by BMAL1, were downregulated, and this change was reversed by the use of ferroptosis inhibitors (Supplementary Fig. 2A, B).

We investigated whether the degradation of BMAL1 took place in AML cells that underwent other types of cell death (apoptosis and necroptosis).Our results showed that BMAL1 was not degraded in apoptosis or necroptosis (Supplementary Fig. 2C). In terms of controls, it was observed that apoptosis inhibitors and necroptosis inhibitors effectively inhibited both apoptosis and necrosis in AML cells, whereas ferroptosis inhibitors did not (Supplementary Fig. 2D,E). Taken together, these results indicate that during the process of ferroptosis in AML cells, RSL3 specifically triggers the degradation of BMAL1 protein.

The proteasomal pathway and autophagy were identified as two distinct mechanisms for the intracellular degradation of proteins. We used inhibitors for each pathway separately to ascertain the degradation mechanism of BMAL1. According to our findings, the proteasomal pathway inhibitor MG132 was unable to prevent the degradation of BMAL1 during RSL3-induced ferroptosis in AML cells (Supplementary Fig. 2F). In contrast, the autophagy inhibitor spautin-1 effectively inhibited its degradation (Supplementary Fig. 2G). Based above discoveries, we have observed that during ferroptosis in AML, the degradation of BMAL1 protein is primarily mediated by autophagy rather than the proteasome pathway. Moreover, our results showed that the ferroptosis inhibitor effectively inhibited the degradation of BMAL1 during RSL3-induced ferroptosis (Supplementary Fig. 2G).

Following that, we examined the impact of BMAL1 degradation during ferroptosis in AML cells. BMAL1 was effectively overexpressed and knocked down in AML cell lines HL60 and MOLM13 (Fig. 1B). Our study demonstrated that the increase in BMAL1 expression led to a notable reduction in cell death [Fig. 2E, F (left)] and a decrease in the production of malondialdehyde (MDA, a marker of lipid peroxidation) [Fig. 2G, H (left)]. These findings indicate that upregulation of BMAL1 has a protective effect against ferroptosis in AML cells. In contrast, our study revealed that stable knockdown of BMAL1 in HL60 and MOLM13 cells restored cell death [Fig. 2E, F (right)] and malondialdehyde (MDA) production [Fig. 2G, H (right)] after treatment with RSL3. These results indicate that the knockdown of BMAL1 increased the sensitivity of AML cells to ferroptosis. Furthermore, the ferroptosis inhibitor was able to reverse the cell death MDA production promoted by BMAL1 knockdown (Supplementary Fig. 3A, C), while apoptosis inhibitors and necroptosis inhibitors were unable to do so (Supplementary Fig. 3B, D). These findings strongly indicate that BMAL1-knockdown-induced cell death is mediated specifically through ferroptosis. Taken together, our study suggests that BMAL1 functions as an inhibitor of ferroptosis in AML cells.

### BMAL1 regulates ferroptosis via HMGB1-GPX4 signaling pathway

Autophagy serves a dual function in maintaining cellular homeostasis. By engaging in controlled self-degradation, autophagy efficiently eliminates detrimental intracellular components, thus safeguarding cells from apoptotic demise (Yang and Klionsky 2010). Nonetheless, when autophagy becomes excessive, it can lead to cell death, including a form known as ferroptosis (Denton and Kumar 2019).The response to ferroptosis mediated by autophagy modulators is context-dependent, including proteins such as BECN1 and HMGB1 (Chen et al. 2021a, b). BECN1, a crucial protein implicated in the initiation of autophagy, assumes a pivotal function in facilitating this process. HMGB1 is an important binding partner of BECN1 that supports the maintenance of autophagy. Cytosolic HMGB1 can interact with BECN1 and direct it to autophagosomes, thereby promoting autophagy and subsequent ferroptosis (Tang et al. 2010). Furthermore, our previous studies have revealed the inhibitory role of BMAL1 in ferroptosis, while the autophagic degradation of BMAL1 enhances the process of ferroptosis. Based on these observations, we hypothesized that BMAL1 might regulate HMGB1 and confer resistance to ferroptosis.

**Fig. 3:**
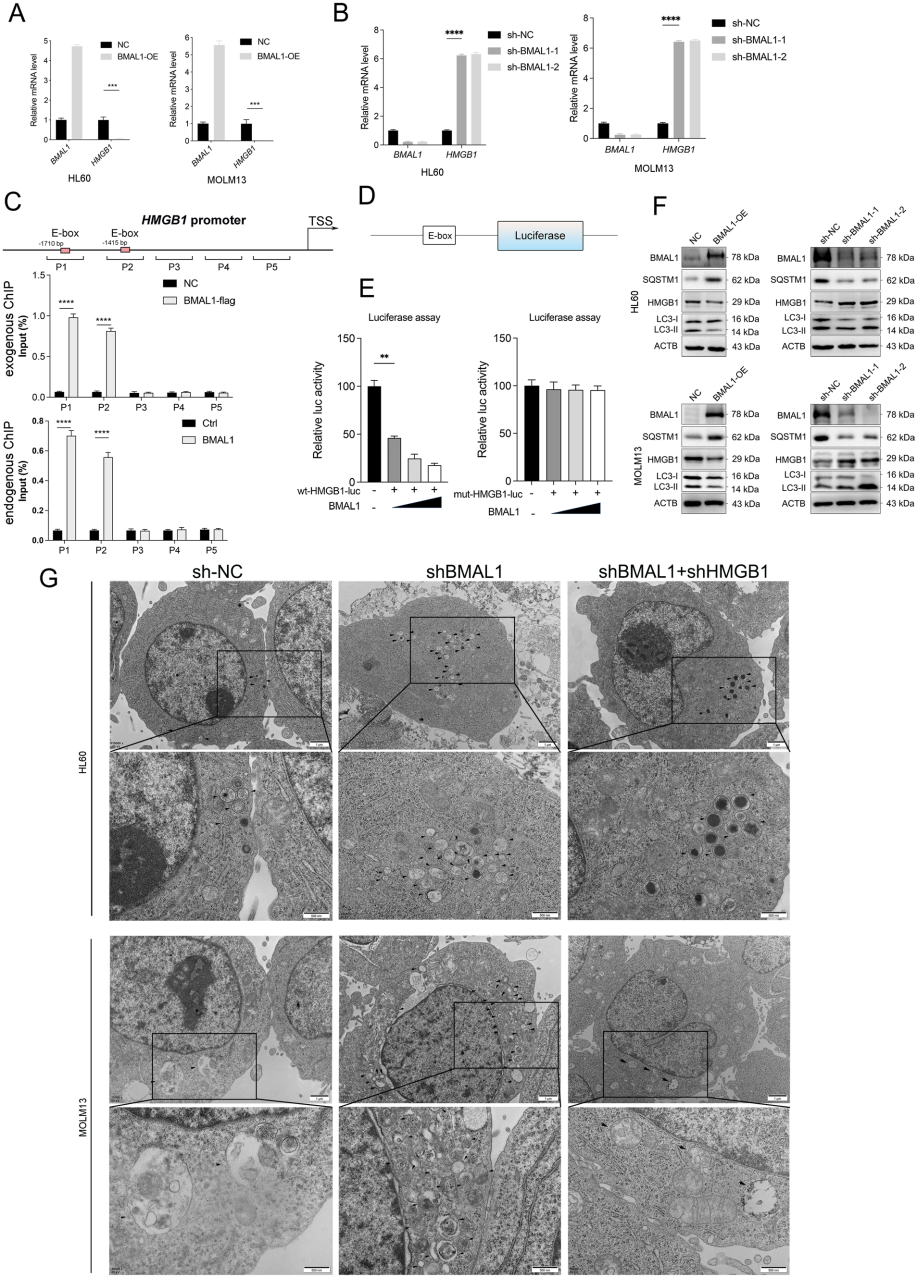
BMAL1 regulates ferroptosis via HMGB1-autophagy-GPX4 pathway.** A** Quantitative polymerase chain reaction (qPCR) analysis of the *HMGB1* mRNAs in both controls and BMAL1-overexpressing HL60 and MOLM13 cells following treatment with RSL3 (1.0 μM) for 24h. **B** Quantitative polymerase chain reaction (qPCR) analysis of the *HMGB1* mRNAs in both controls and BMAL1-depleted HL60 and MOLM13 cells following treatment with RSL3 (1.0 μM) for 24h. **C** ChIP-qPCR analysis was used to determine the binding affinity of BMAL1 to HMGB1 promoter regions.293T cells were transfected with BMAL1 overexpression plasmid that ChIP-qPCR with Flag(left) were performed as the control(left). 293T cells were not transfected with BMAL1 overexpression plasmid that ChIP-qPCR with IgG (left) were performed as the control(right). **D** A schematic representation of the constructed 5'UTR region of HMGB1 containing E-box elements. **E** Luciferase reporter gene constructs were cotransfected with BMAL1 overexpression plasmid in 293T cells, and reporter gene activity was measured after 48 h by a dual luciferase assay. The relative value in 293T cells cotransfected with vector was set to 100%. **F** Western blot analysis of the indicated proteins in both controls, BMAL1-overexpressing and BMAL1-depleted HL60 and MOLM13 cells following treatment with RSL3 (1.0 μM) for 24 h. **G** The number changes of autophagosomes of both controls, BMAL1-depleted and BMAL1-HMGB1-depleted HL60 and MOLM13 were detected by transmission electron microscopy (TEM). n.s. (no significance), Statistical significance in (**A**–**C**) was calculated by two-way ANOVA with Dunnett’s multiple comparison test, and by one-way ANOVA with Dunnett’s multiple comparison test for (**E**).*p < 0.05; **p < 0.01; ***p < 0.001. ****p < 0.0001

To explore how BMAL1 regulates HMGB1 during ferroptosis in AML cells, we conducted various in vitro assays using HL60 and MOLM13 cells with BMAL1 overexpression or knockdown. To investigate the direct regulatory relationship between BMAL1 and HMGB1 in the context of ferroptosis, we utilized quantitative polymerase chain reactions (qPCRs). In our investigation, we observed that upregulation of the BMAL1 in HL60 and MOLM13 cells resulted in a downregulation of *HMGB1* mRNA levels (Fig. 3A). Conversely, when we knocked down BMAL1, it led to an upregulation of *HMGB1* mRNA levels (Fig. 3B).

As a transcription factor, BMAL1 mediates its regulatory function by interacting with E-box elements. Upon careful examination of the Eukaryotic Promoter Database, it was discovered that the promoter region of HMGB1 does not contain any E-box elements. After reviewing the literature and being inspired by related studies, we hypothesized that the transcription factor BMAL1 might have exerted transcriptional regulation on HMGB1 through binding to the upstream region of the transcription start site (TSS) of HMGB1. After a comprehensive literature review, we validated the existence of E-box elements within 2000-base pair region upstream of the TSS of HMGB1.The sequence is provided as supporting data.

To determine whether BMAL1 could bind to the E-box elements within the 5'UTR region of HMGB1, we performed chromatin immunoprecipitation experiments. The results of our study confirmed our hypothesis, providing evidence that BMAL1 can indeed bind to the E-box elements within the upstream of the TSS of HMGB1 (Fig. 3C). Moreover, reporter gene analysis substantiated that BMAL1 trans-repressed HMGB1 during RSL3-induced ferroptosis (Fig. 3D, E). And after the mutation that disrupts the binding site of BMAL1 on HMGB1, BMAL1 could not inhibit the transcription of HMGB1. Additionally, western blot results depicted that the overexpression of BMAL1 downregulated the expression of HMGB1 and attenuated autophagy (Fig. 3F, G). Conversely, stable knockdown of BMAL1 upregulated HMGB1 expression and enhanced autophagy (Fig. 3F, G). In conclusion, the above results indicate that BMAL1 can transcriptionally regulate HMGB1, thereby influencing autophagy.

**Fig. 4:**
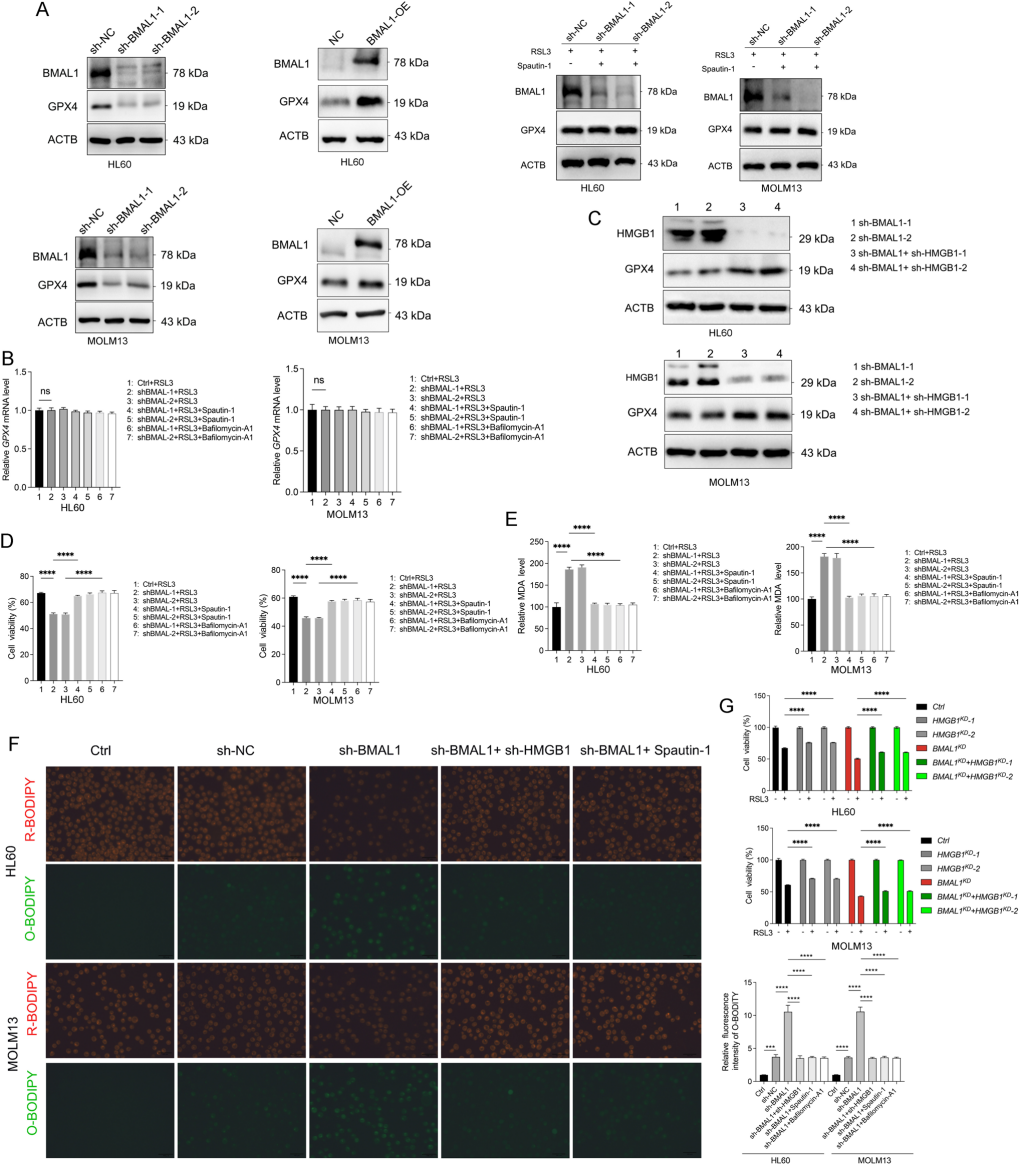
BMAL1 regulates ferroptosis via HMGB1-GPX4 pathway**. A** Western blot analysis of the indicated proteins in both controls, BMAL1-depleted and BMAL1-overexpression HL60 and MOLM13 cells following treatment with RSL3 (1.0 μM) for 24h in the absence or presence of spautin-1 (5.0 μM), bafilomycin A1 (50.0 nM) for 24 h. **B** Quantitative polymerase chain reaction (qPCR) analysis of the *GPX4* mRNAs in the indicated gene knockdown HL60 and MOLM13 cells after treatment with RSL3 (1.0 μM) for 24h. **C** Western blot analysis of the indicated proteins in BMAL1-depleted and both BMAL1, HMGB1-depleted HL60 and MOLM13 cells following treatment with RSL3 (1.0 μM) for 24h. **D** Cell viability of both control and BMAL1-depleted HL60 and MOLM13 following treatment with RSL3(1.0 μM) in the absence or presence of spautin-1 (5.0 μM), bafilomycin A1 (50.0 nM) for 24 h. **E** Analysis of MDA levels in both controls and BMAL1-depleted HL60 and MOLM13 cells following treatment with RSL3(1.0 μM) in the absence or presence of spautin-1 (5.0 μM), bafilomycin A1 (50.0 nM) for 24 h. **F** Analysis of lipid peroxidation in the indicated gene knockdown HL60 and MOLM13 cells after treatment with RSL3 (1.0 μM) for 24 h. **G** Analysis of cell death in the indicated gene knockdown HL60 and MOLM13 cells after treatment with RSL3 (1.0 μM) for 24 h. Data represents the mean ± SD from three independent experiments; n.s. (no significance), Statistical significance in (**B**). **D**–**F** was calculated by one-way ANOVA with Dunnett’s multiple comparison test, and by two-way ANOVA with Tukey’s multiple comparison test for (**G**).*p < 0.05; **p < 0.01; ***p < 0.001. ****p < 0.0001

Certain selective types of autophagy, as described in the introduction section, involves the degradation of specific ferroptosis-inhibitory proteins, which can facilitate ferroptosis. We observed that knockdown of BMAL1 led to upregulated autophagy, which facilitated the degradation of GPX4 (Fig. 4A, Supplementary Fig. 4A). Conversely, overexpression of BMAL1 inhibited GPX4 degradation compared to control cells following treatment with RSL3 (Fig. 4A, Supplementary Fig. 4A). To further validate our results and hypotheses, we assessed the mRNA level of GPX4. Our results suggested that knocking down BMAL1 or inhibiting autophagy has no significant effect on the mRNA levels of GPX4 (Fig. 4B).

To determine whether BMAL1 regulates ferroptosis in AML cells through the HMGB1-GPX4 pathway, we treated AML cells with stably depletion of HMGB1 and BMAL1 with RSL3. Our results demonstrated that depletion of HMGB1 inhibited GPX4 degradation in BMAL1-depleted AML cells (Fig. 4C). In addition, the use of spautin-1 and bafilomycin A1 (autophagy inhibitors) suppressed autophagy and reversed the degradation of GPX4 promoted by BMAL1 knockdown (Fig. 4A, Supplementary Fig. 4B). Our findings also showed that the inhibition of autophagy limited cell death (Fig. 4D), the production of MDA (Fig. 4E) and lipid peroxidation (Fig. 4F, Supplementary Fig. 4C) in both HL60 and MOLM13 cells after treatment with RSL3. These results suggest that HMGB1 depletion and treatment with autophagy inhibitors rescued GPX4 degradation and ferroptosis.

To further validate our observations, we conducted rescue experiments. Our results demonstrated that knockdown of HMGB1 with two shRNAs (Supplementary Fig. 4D) not only resulted in reduced MDA production (Supplementary Fig. 4E) and lipid peroxidation (Fig. 4F), but also reversed cell death (Fig. 4G), and autophagy (Fig. 3F) of HL60 and MOLM13 cells after treated with RSL3. In summary, the above results indicate that BMAL1 can inhibit ferroptosis through the HMGB1-GPX4 pathway.

### Targeting BMAL1 sensitizes AML cells to targeted agents’ treatment

Survey data indicates that cancer has risen to become the second most common, and in some countries, the primary cause of mortality worldwide (Soerjomataram and Bray 2021). Therefore, it is crucial to continue efforts in finding effective novel therapies to improve survival rates and reduce long-term morbidity. The advancement of novel pharmaceuticals in the field of oncology, particularly for AML, presents substantial hurdles (Döhner et al. 2021). Compared to old drugs used in novel treatments or in combination with other therapeutic agents, the development of new drugs is more difficult and takes longer (Short et al. 2020). Taking into account the role of BMAL1 in the pathogenesis of AML, our hypothesis suggests a potential association between BMAL1 and chemotherapy resistance in AML.

To ascertain the involvement of BMAL1 in chemoresistance of AML,we utilized AML cells overexpressing BMAL1 and treated them with various therapeutic drugs for AML. After examining a range of therapeutic drugs,we did not observe high expression of BMAL1 resulted in resistance of AML cells to commonly used chemotherapeutic drugs such as doxorubicin and daunorubicin. But our results confirmed that AML cells overexpressing BMAL1 exhibited drug resistance to dasatinib, venetoclax and sorafenib.

**Fig. 5:**
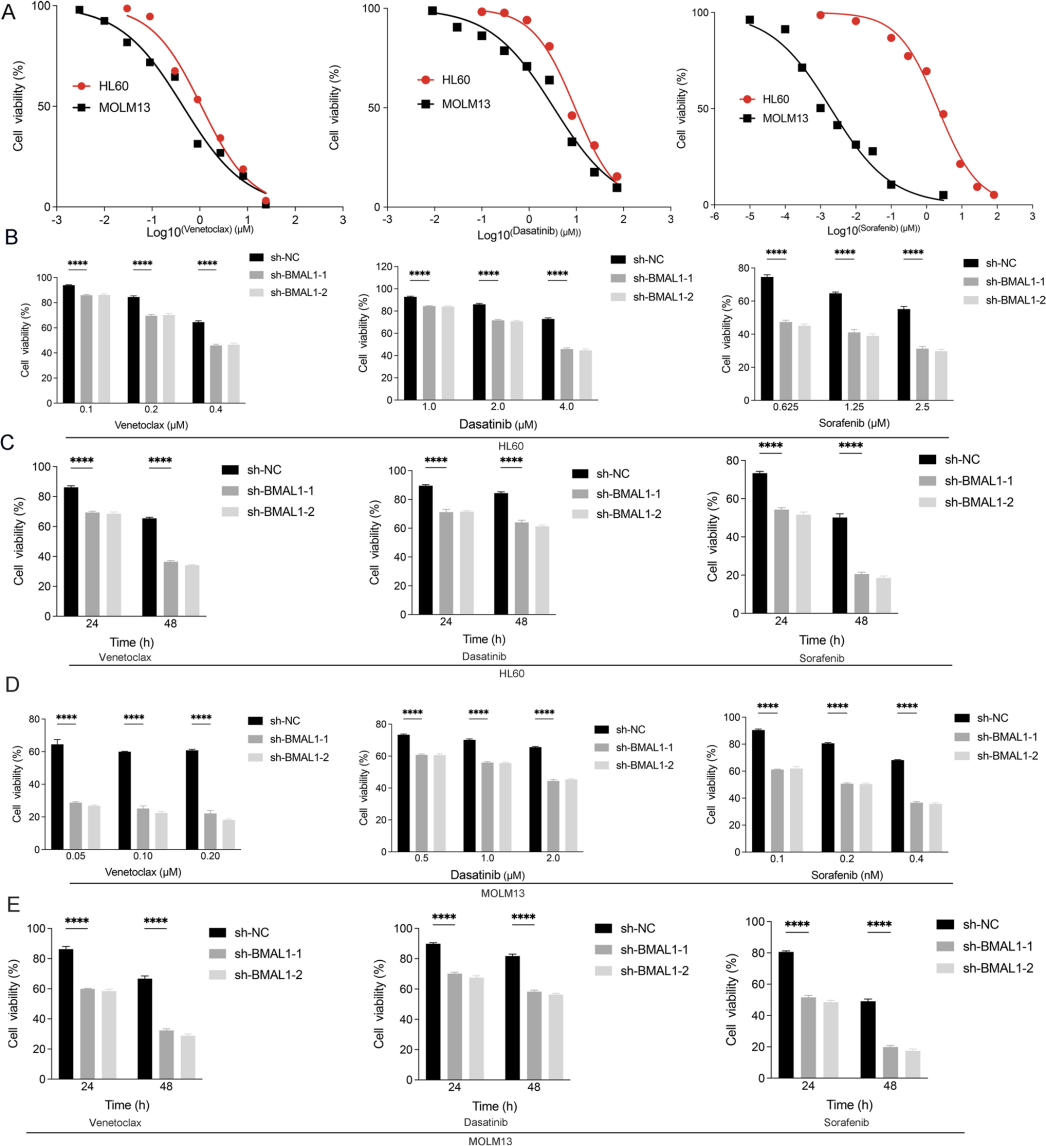
Targeting BMAL1 sensitizes AML cells to targeted agents. **A** Cell viability of HL60 and MOLM13 cells following treatment with venetoclax, dasatinib and sorafenib at the indicated concentration for 24 h. **B** Cell viability of both control and BMAL1-depleted HL60 cells treated with venetoclax, dasatinib and sorafenib at different concentrations as indicated for 24 h. **C** Cell viability of both control and BMAL1-depleted HL60 cells treated with venetoclax (0.2 μM), dasatinib (2.0 μM) and sorafenib (0.3 μM) at different time points as indicated. **D** Cell viability of both control and BMAL1-depleted MOLM13 cells treated with venetoclax, dasatinib and sorafenib at different concentrations for 24 h. **E** Cell viability of both control and BMAL1-depleted MOLM13 cells treated with venetoclax (0.02 μM), dasatinib (0.1 μM) and sorafenib (0.2 nM) at different time points as indicated. Data represents the mean ± SD from three independent experiments; n.s. (no significance), Statistical significance in (**B**–**E**) was calculated by two-way ANOVA with Dunnett’s multiple comparison test.*p < 0.05; **p < 0.01; ***p < 0.001. ****p < 0.0001

To corroborate our observations, we initially investigated the responsiveness of AML cells to venetoclax, dasatinib, and sorafenib. The dose–response curve indicated that HL60 and MOLM13 cells were sensitive to venclexta, dasatinib, and sorafenib, with MOLM13 showing greater sensitivity than HL60 cells (Fig. 5A). We then introduced BMAL1 overexpression in HL60 and MOLM13 cells, which resulted in reduced cell death after treatment with venetoclax, dasatinib, and sorafenib (Supplementary Fig. 5A-D). In contrast, stable knockdown of BMAL1 restored sensitivity to venetoclax, dasatinib, and sorafenib in HL60 and MOLM13 cells (Fig. 5B–E).

**Fig. 6:**
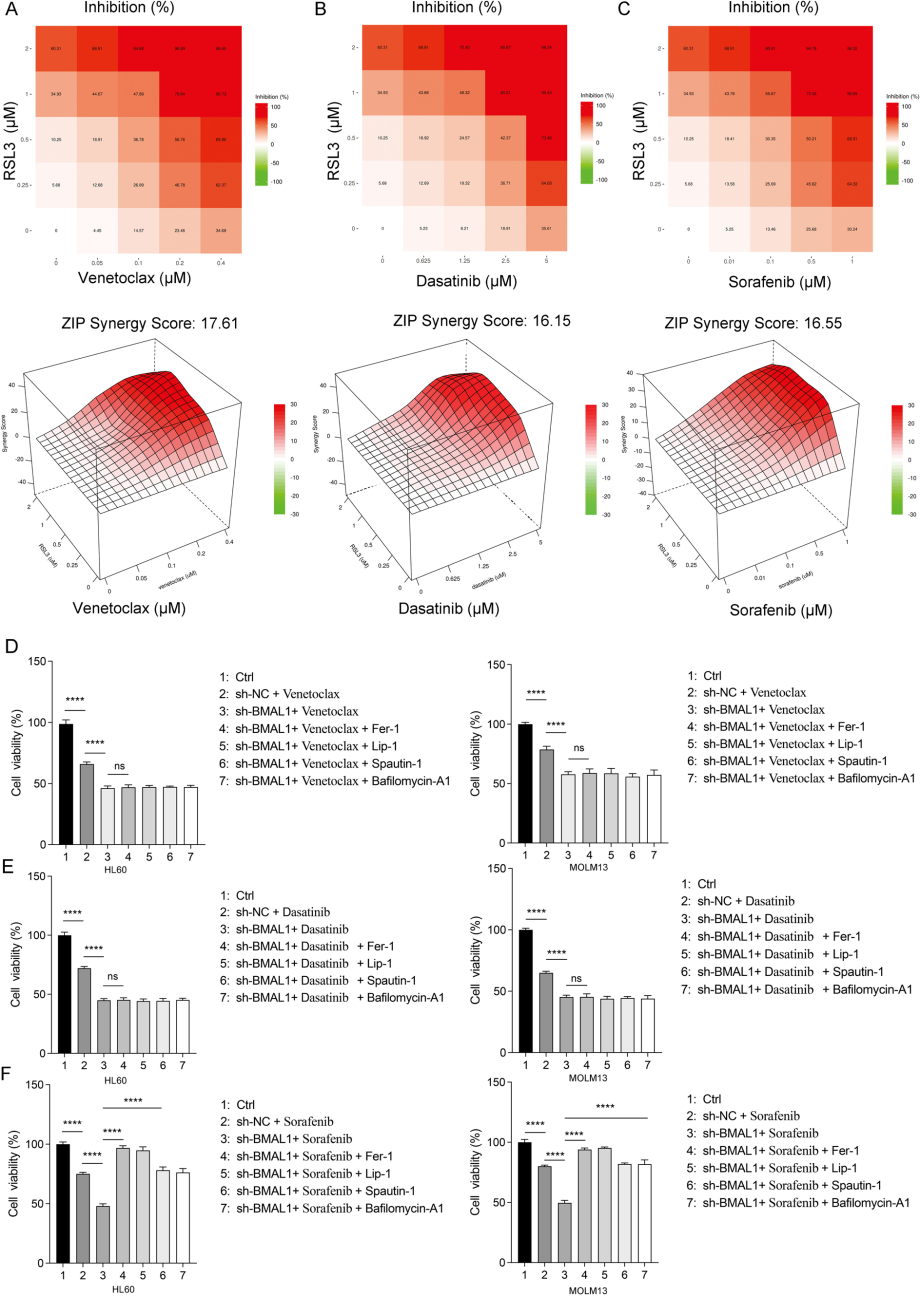
Synergistic effects between targeted agents and RL3. **A** Cell viability of HL60 cells treated with venetoclax and RSL3 at different concentrations as indicated for 24 h. ZIP synergy scores were performed by SynergyFinder. A score ≥ 10 indicates a strong synergistic effect between two drugs. **B** Cell viability of HL60 cells treated with dasatinib and RSL3 at different concentrations as indicated for 24 h. **C** Cell viability of HL60 cells treated with sorafenib and RSL3 at different concentrations as indicated for 24 h. **D** Cell viability of both control and BMAL1-depleted HL60 and MOLM13 cells following treatment with venetoclax (HL60: 0.4 μM, MOLM13: 0.02 μM) in the absence or presence of ferrostatin-1 (0.5 μM), liproxstatin-1 (0.5 μM), spautin-1 (5.0 μM) and bafilomycin A1 (50.0 nM) for 24 h. **E** Cell viability of both control and BMAL1-depleted HL60 and MOLM13 cells following treatment with dasatinib (HL60: 4.0 μM, MOLM13: 2.0 μM) in the absence or presence of ferrostatin-1 (0.5 μM), liproxstatin-1 (0.5 μM), spautin-1 (5.0 μM) and bafilomycin A1 (50.0 nM) for 24 h. **F** Cell viability of both control and BMAL1-depleted HL60 and MOLM13 cells following treatment with sorafenib (HL60: 0.625 μM, MOLM13: 0.2 μM) in the absence or presence of ferrostatin-1 (0.5 μM), liproxstatin-1 (0.5 μM), spautin-1 (5.0 μM) and bafilomycin A1 (50.0 nM) for 24 h. Data represents the mean ± SD from three independent experiments; n.s. (no significance), Statistical significance in (**D–F**) was calculated by one-way ANOVA with Tukey’s multiple comparison test.*p < 0.05; **p < 0.01; ***p < 0.001. ****p < 0.0001

Considering the promotion of BMAL1 degradation by RSL3-induced ferroptosis, leading to decreased BMAL1 protein levels, we hypothesized a potential synergistic effect between RSL3 and chemotherapeutic agents. Subsequently, we evaluated the combined effects of these drugs on cell death. Our results demonstrated a synergistic interaction between RSL3 and venetoclax, dasatinib, and sorafenib (Fig. 6A–C). SynergyFinder (a tool for professional analysis and visualization of multidrug combination response data) was employed to assess drug synergy scores. Our findings revealed a synergistic promotion of cell death between RSL3 and venetoclax, dasatinib, and sorafenib (Fig. 6A–C).

Lastly, we investigated whether the knockdown of BMAL1 promoted the cytotoxicity of venetoclax, dasatinib, and sorafenib through ferroptosis. Our results demonstrated that BMAL1 knockdown-mediated cell death induced by venetoclax and dasatinib was not ferroptosis or autophagic cell death (Fig. 6D–E). In contrast, the cell death induced by sorafenib was identified as ferroptosis (Fig. 6F). As autophagy plays a significant role in the process of ferroptosis (Chen et al. 2021a, b), the inhibition of autophagy also reduced cell death (Fig. 6F). Consistent with our findings, previous studies have reported that sorafenib can induce ferroptosis in various tumor cells (Gao et al. 2021; Byun et al. 2022; Xu et al. 2023). Furthermore, it was observed that overexpression of BMAL1 resulted in decreased levels of both HMGB1 protein and mRNA after treatment with venetoclax, dasatinib, and sorafenib (Supplementary Fig. 5E–F). No significant changes in the mRNA levels of GPX4 were observed(Supplementary Fig. 5E), but the overexpression of BMAL1 reduced the degradation of GPX4 after treatment with sorafenib (Supplementary Fig. 5F). These results were consistent with our aforementioned findings.

The above results indicate that knockdown of BMAL1 can render AML cells more sensitive to anticancer drugs, including venetoclax, dasatinib, and sorafenib, suggesting its potential as a novel therapeutic target for AML.

### BMAL1 regulates ferroptosis and anticancer agents’ efficacy in *vivo*

**Fig. 7:**
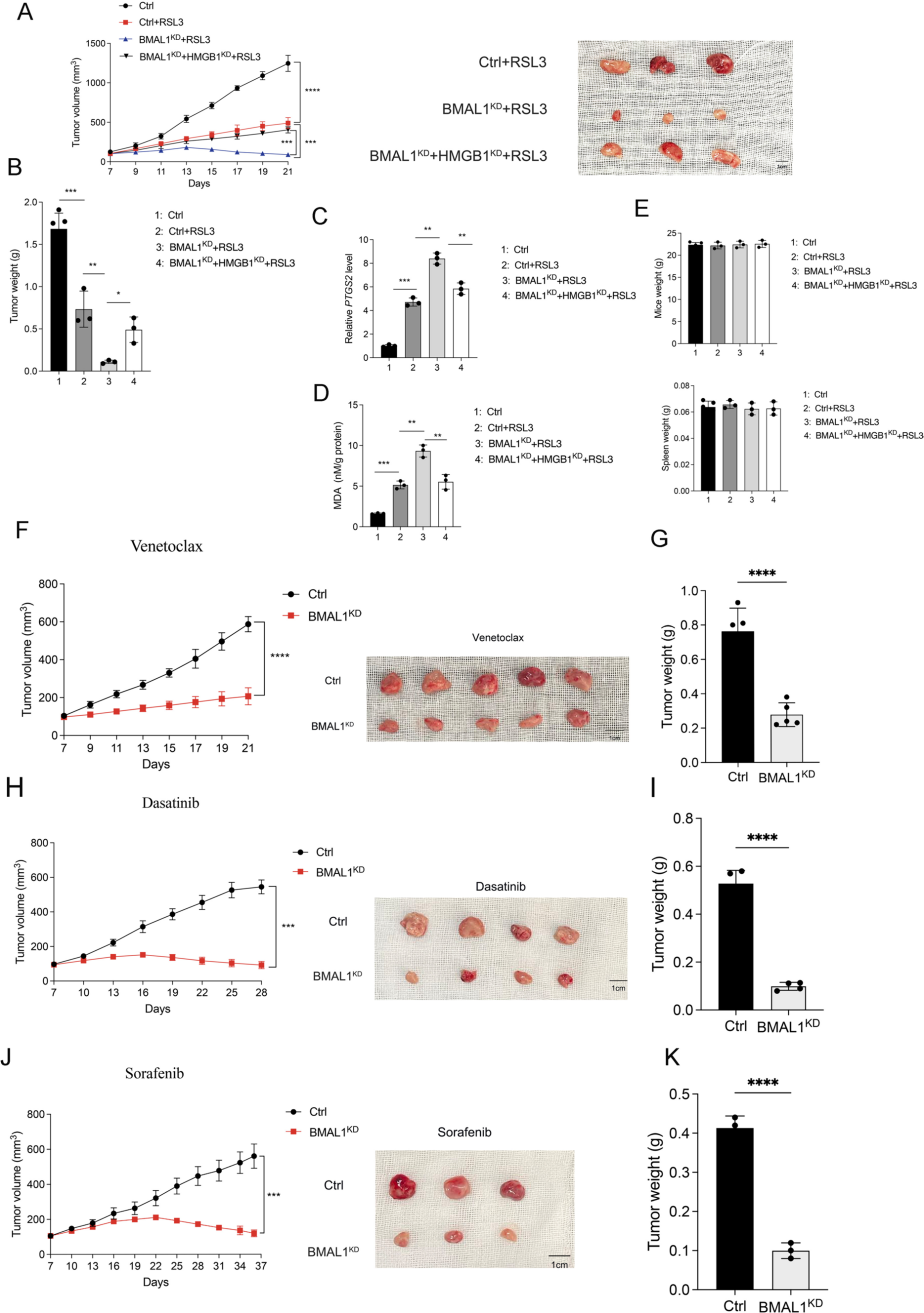
BMAL1 regulates ferroptosis and anticancer agents’ efficacy in *vivo. A–B* Volumes and weight of subcutaneous tumors in indicated mice. Athymic nude mice were injected subcutaneously with the indicated HL60 cells for 7 days and then treated with RSL3 (30 mg/kg; intraperitoneally, once every other day) at day 7 for 2 weeks. Tumor volumes were calculated once every other day (*n* = 3 mice per group, *p < 0.05). **C–D** *PTGS2* mRNA and MDA level in isolated tumors at day 14 after treatment were assayed. **E** The weight of indicated mice and spleen. **F–G** Volumes and weight of subcutaneous tumors in indicated mice following treatment with venetoclax. Athymic nude mice were injected subcutaneously with the indicated HL60 cells for 7 days and then treated with venetoclax (50 mg/kg; recipient mice were treated with venetoclax (daily by oral gavage) at day 7 for 2 weeks. Tumor volumes were calculated once every other day (*n* = 5 mice per group, *p < 0.05 versus ctrl + BMAL1^KD^ group). **H–I** Volumes and weight of subcutaneous tumors in indicated mice following treatment with dasatinib. Athymic nude mice were injected subcutaneously with the indicated HL60 cells for 7 days and then treated with dasatinib (50 mg/kg; recipient mice were treated with dasatinib (daily by oral gavage) at day 7 for 3 weeks. Tumor volumes were calculated once every three days (*n* = 4 mice per group, *p < 0.05 versus ctrl + BMAL1^KD^ group). **J–K** Volumes and weight of subcutaneous tumors in indicated mice following treatment with sorafenib. Athymic nude mice were injected subcutaneously with the indicated HL60 cells for 7 days and then treated with sorafenib (30 mg/kg; recipient mice were treated with sorafenib (daily by oral gavage) at day 7 for 4 weeks. Tumor volumes were calculated once every three days (*n* = 3 mice per group, *p < 0.05 versus ctrl + BMAL1^KD^ group). Data represents the mean ± SD from three to five independent experiments; n.s. (no significance), Statistical significance in (**A**, **F**–**J**) was calculated by two-way ANOVA with Dunnett’s multiple comparison test, by one-way ANOVA with Dunnett’s multiple comparison test for (**B**–**E**),and by two-tailed unpaired t test for (**G**, **I**, **K**).*p < 0.05; **p < 0.01; ***p < 0.001. ****p < 0.0001

In order to study the in *vivo* mechanism by which BMAL1 regulates ferroptosis through HMGB1, we utilized subcutaneous tumor implantation to establish AML tumor models. After the tumor model was established on the seventh day, the mice underwent treatment with RSL3, which was given every other day for two weeks. The therapeutic response indicated a significant decrease in tumor volume and weight among the group of mice with BMAL1 knockdown compared to the control group (Fig. 7A, B). Subsequently, tumor tissue was collected for relevant analyses. The results of in *vivo* detection markers, including *PTGS2* mRNA levels and MDA quantification, suggested that knocking down BMAL1 increased tumor cell susceptibility to ferroptosis (Fig. 7C, D). In contrast, mice with both BMAL1 and HMGB1 knockdown cells showed resistance to RSL3 treatment (Fig. 7A, B). Detection of *PTGS2* expression level and MDA quantification confirmed that knocking down BMAL1 promoted ferroptosis.

Simultaneously, knocking down HMGB1 reversed the BMAL1 knockdown-induced promotion of ferroptosis (Fig. 7C, D). Importantly, no significant change in body weight or spleen weight (Fig. 7E) of host mice were observed, indicating no adverse health consequences of tumor inoculation. In summary, the results of the animal experiments provide evidence supporting the role of BMAL1 in suppressing ferroptosis in AML cells through the involvement of HMGB1.

In order to explore the influence of BMAL1 in *vivo* on the responsiveness of AML cells to venetoclax, dasatinib, and sorafenib, we conducted AML tumor modeling by subcutaneously injecting mice with either stable BMAL1-knockout cells or cells transfected with an empty vector. Subsequent to the completion of tumor modeling, the mice were individually administered with venetoclax, dasatinib, and sorafenib.The administration of venetoclax, dasatinib, and sorafenib demonstrated significant efficacy in reducing tumor size in mice harboring BMAL1 knockdown cells.

Within the cohorts receiving venetoclax treatment, the administration of this therapeutic agent resulted in a substantial reduction in tumor size among mice harboring BMAL1 knockdown cells. This phenomenon was observed from day 13 to day 21. Our findings demonstrate that stable BMAL1 depletion led to smaller tumors in mice (Fig. 7F, G). By the 21st day, the mice that were injected with BMAL1-depleted cells demonstrated a tumor volume measuring 207.0 ± 39.8 mm^3, whereas the control group exhibited a larger tumor volume of 587.9 ± 35.8 mm^3 (Fig. 7F). Concerning the weight of the tumors, there was a notable decrease in tumor weight in the BMAL1 knockdown group. The tumor weight in the group of mice with BMAL1 knockdown was measured to be 0.28 ± 0.06 g, whereas the control group exhibited a notably higher tumor weight of 0.76 ± 0.12 g (Fig. 7G).

In the dasatinib treatment groups, the administration of dasatinib led to a considerable decrease in tumor size in mice carrying BMAL1 knockdown cells. From day 16 to day 28, a noticeable difference in tumor size was observed, indicating that dasatinib treatment was effective in reducing tumor growth in these mice (Fig. 7H, I). On day 28, the mice that were injected with BMAL1-depleted cells showed a significantly smaller tumor volume of 92.2 ± 17.9 mm^3, in contrast to the control group, which exhibited a larger tumor volume of 544.9 ± 34.6 mm^3 (Fig. 7H). Moreover, when reaching the endpoint of the observation, the tumor weight in mice subjected to BMAL1 knockdown was notably reduced compared to the tumor weight in mice without BMAL1 knockdown. Specifically, the control group exhibited an average tumor weight of 0.53 ± 0.05 g, whereas tumors that derived from mice with BMAL1-depleted cells weighed only 0.10 ± 0.01 g (Fig. 7I).

In the sorafenib treatment groups, the administration of sorafenib effectively resulted in a significant reduction in tumor size in mice that carried BMAL1 knockdown cells compared to the control group. This reduction was observed from day 25 to day 37 (Fig. 7J, K). The outcomes of our study demonstrated that mice receiving stable BMAL1-depleted cells exhibited significantly reduced tumor sizes. Specifically, on day 37, the group of mice injected with BMAL1-depleted cells displayed a tumor volume of 119.4 ± 15.8 mm^3, while the control group exhibited a much larger tumor volume of 560.5 ± 56.7 mm^3 (Fig. 7J). Moreover, it was observed that tumors originating from mice with BMAL1-depleted cells exhibited a considerably lower weight.The control group displayed an average tumor weight of 0.41 ± 0.02 g, whereas tumors derived from mice with BMAL1-depleted cells weighed 0.10 ± 0.02 g, indicating a significant reduction in tumor mass (Fig. 7K).

Remarkably, we detected no notable alterations in the body weight and spleen weight (Supplementary Fig. 6A–C) of the recipient mice, signifying the absence of unfavorable health effects caused by tumor inoculation. These cumulative findings strongly propose that BMAL1 functions as a mediator of venetoclax, dasatinib, and sorafenib resistance in *vivo* within AML.

## Discussion

**Fig. 8:**
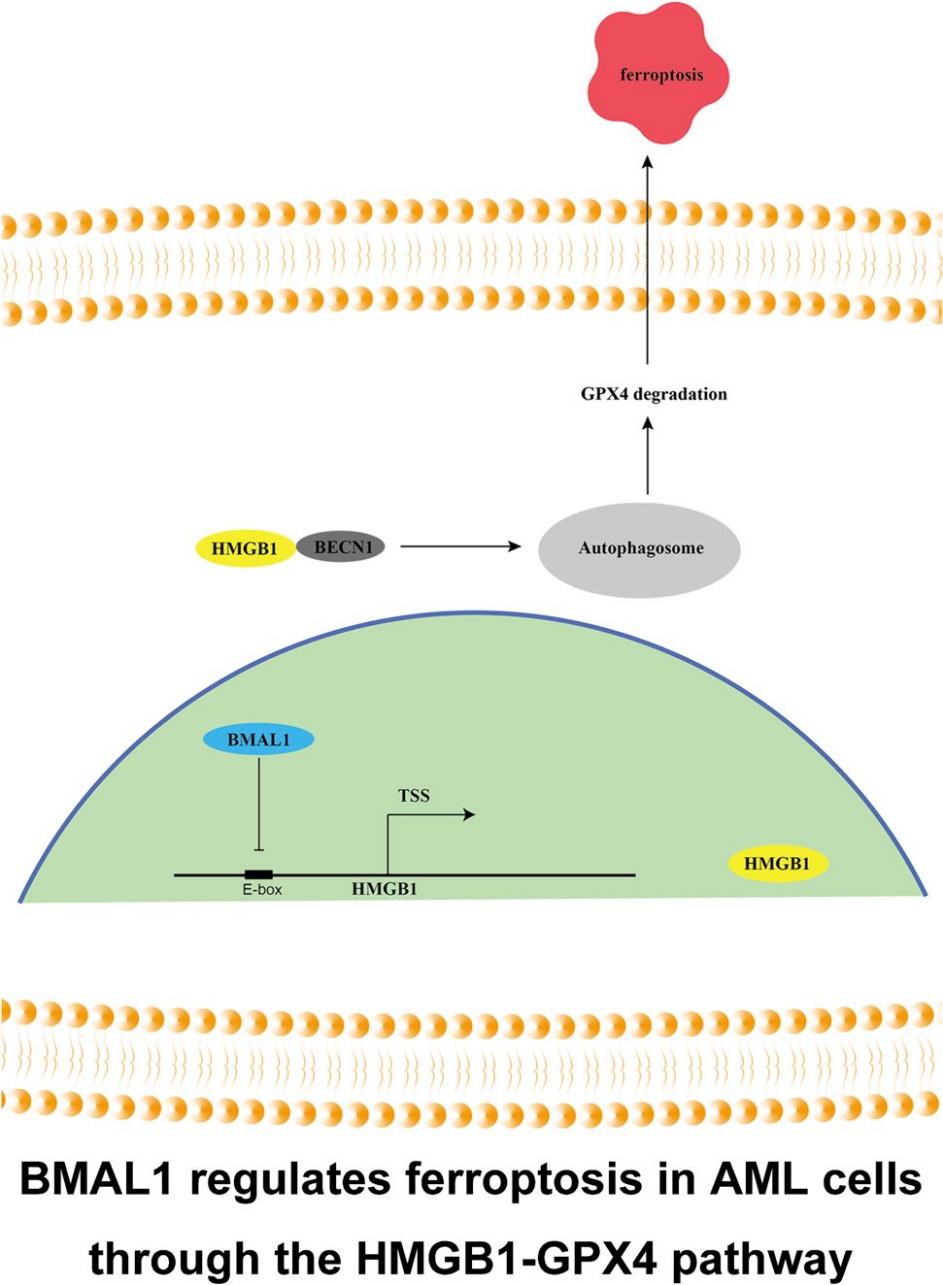
Schematic summary of the role of BMAL1 in the regulation of HMGB1 and ferroptosis

The concept of ferroptosis was first proposed in 2012 by Dr. Brent R Stockwell (Dixon et al. 2012), and it is triggered by the toxic accumulation of lipid peroxides on the cell membrane. Extensive research has demonstrated the significant role of ferroptosis in various organ-related diseases, including the lungs, liver, kidneys, heart, skeletal system, pancreas, and gastrointestinal tract. It is closely associated with the pathological and physiological processes of neurodegenerative diseases, ischemia–reperfusion injury, diabetes, and tumors (Qiu et al. 2020). Furthermore, ferroptosis plays different roles in different diseases, providing a highly promising new approach for the treatment of many diseases by inhibiting or promoting ferroptosis to slow down disease progression (Jiang et al. 2021). In addition, ferroptosis has been confirmed to be associated with multiple tumor signaling pathways, such as P53, HIF, PI3K, and JUN (Tang, et al. 2021). Approved drugs like ciclopirox (Lai et al. 2016), sorafenib (Louandre et al. 2013), dopamine (Wang et al. 2016), and statins (Viswanathan et al. 2017) have been found to promote or inhibit ferroptosis. Currently, ferroptosis has generated immense interest in the field of cancer research, not only because it represents a unique form of cell death but also due to its distinct biochemical, genetic, and mechanistic characteristics compared to other forms of cell death. As a transcription factor, BMAL1 not only impacts the occurrence, progression, and metastasis of tumors, but also correlates with the sensitivity of tumor cells to chemotherapeutic drugs (Miro et al. 2023). The degradation of BMAL1 mediated by autophagy promotes lipid peroxidation, ROS generation, and ultimately leads to the occurrence of ferroptosis (Yang et al. 2019). Therefore, BMAL1 emerges as a potential target in investigating tumor therapeutic strategies aimed at ferroptosis. HMGB1 plays a role in various diseases and pathological processes (Chen et al. 2023b, a). Moreover, HMGB1 also plays an important role in autophagy. However, the understanding of the role and relationship of BMAL1 and HMGB1 in ferroptosis is currently limited. Based on our previous research on BMAL1 and HMGB1, this study investigates the role of BMAL1 in AML and its mechanism of regulating ferroptosis through HMGB1 (Fig. 8).

In this study, it was found that BMAL1 was differentially expressed in 14 types of tumors based on the TCGA data. Distinguishing from the other 13 types, only AML showed higher expression levels of BMAL1 than normal tissues. This may suggests the unique role of BMAL1 in AML. Furthermore, elevated expression of BMAL1 is strongly correlated with unfavorable patient outcomes in individuals diagnosed with AML. In accordance with our discoveries in AML, a previous bioinformatics analysis of AML also showed higher BMAL1 expression compared to matched normal adjacent tissues (Yin et al. 2022). The CLOCK and BMAL1 form a heterodimeric core transcription factor complex, constituting the central oscillator of the circadian clock. Their functions are intricately interconnected. Recent one study has revealed that CLOCK exerts a tumor-promoting effect on glioblastoma progression via the POSTN-TBK1 signaling pathway (Pang et al. 2023). There has also been a study that demonstrates the inhibitory effect of CLOCK on glioblastoma through the OLFML3-HIF1α-LGMN pathway (Xuan et al. 2022). Additionally, BMAL1 and CLOCK can impact the cell cycle of hepatocellular carcinoma cells by regulating the expression of Wee1 and P21, thereby contributing to tumor progression (Qu et al. 2023). Based on the integration of previous research findings and our own findings, we proposed a hypothesis that BMAL1 exerts a facilitative role in the progression of AML. Therefore, we further investigated the role of BMAL1 in AML. Our research results showed that overexpression of BMAL1 promoted the proliferation of AML cells HL60 and MOLM13, while knockdown of BMAL1 arrested the cell cycle and slowed down cell proliferation in AML cells. These results further confirm that BMAL1 may play a promoting role in the disease progression of AML patients. Furthermore, when BMAL1-depleted HL60 cells were injected into BALB/c mice, it provided further evidence to support the involvement of BMAL1 in promoting tumor growth in *vivo*. Previous reports have also demonstrated the essential role of BMAL1 in leukemia cell growth without causing hematopoietic defects, suggesting that BMAL1 genes could be effective therapeutic targets in AML (Puram et al. 2016). In summary, BMAL1 exerts a positive promoting effect on the growth of AML cells, and its high expression may indicate an adverse outcome. Therefore, BMAL1 has the potential to be targeted for the development of future AML therapies. Further exploration of the prognostic value of BMAL1 and the mechanism underlying its high expression in AML is necessary.

Our findings provide evidence that BMAL1 could regulate ferroptosis in AML cells through the HMGB1-GPX4 pathway. BMAL1 acts as a suppressor of ferroptosis. Consequently, the autophagic degradation of BMAL1 can facilitate the occurrence of ferroptosis. BECN1, a BH3-only protein, forms a complex with other proteins to promote the formation of autophagic vesicles, thereby initiating autophagy (Dikic et al. 2018). HMGB1 has multiple functions, and the functions of HMGB1 vary depending on its subcellular localization (Chen et al. 2023b, a). Cytosolic HMGB1 can bind to BECN1, inducing autophagosome formation and promoting autophagy (Kang et al. 2010; Tang et al. 2010). The findings of this study suggested that BMAL1 inhibited autophagy and GPX4 degradation by suppressing the transcriptional expression of HMGB1, thereby suppressing ferroptosis. In our study, we first observed a reduction in both protein and mRNA levels of HMGB1 when BMAL1 was overexpressed. Conversely, the knockdown of BMAL1 resulted in an elevation of HMGB1 at both protein and mRNA levels. Furthermore, BMAL1 functions as a transcription factor. Based on these findings, we proposed a hypothesis that BMAL1 plays a regulatory role in the transcriptional expression of HMGB1. The transcriptional regulation of BMAL1 involves its interaction with E-box elements located in the promoter region. However, it was observed that the promoter region of HMGB1 does not contain any E-box elements. Consequently, we hypothesized the presence of E-box elements in the downstream region, particularly in the 5'UTR region, of the HMGB1 promoter. Subsequent sequence analysis provided confirmation of the existence of E-box elements within the 5'UTR region of HMGB1. Consequently, chromatin immunoprecipitation (ChIP) experiments and luciferase assays were conducted. Both exogenous and endogenous ChIP experiments confirmed the binding of BMAL1 to the DNA sequence of HMGB1. The results of luciferase assays demonstrated that BMAL1 could suppress the transcription of HMGB1 containing E-box elements. However, when the E-box elements were mutated, BMAL1 lost its ability to inhibit the transcription of HMGB1. In conclusion, BMAL1 can inhibit the transcription of HMGB1, reducing HMGB1 protein levels and subsequently leading to a decrease in autophagy activity. lipid peroxidation and/or decreased cellular antioxidant capacity can result in ferroptosis. Knockdown of BMAL1 promotes autophagy. The ferroptosis suppressor GPX4 is one of the most vital cellular antioxidant factors (Weaver and Skouta 2022). Numerous studies have demonstrated that degradation of GPX4 can facilitate the occurrence of ferroptosis (Xie et al. 2023). Therefore, we speculated that the knockdown of BMAL1 resulted in enhanced autophagy, promoted GPX4 degradation, subsequently leading to a decrease in cellular antioxidant capacity, ultimately facilitating ferroptosis. Through experiments, we observed that overexpression of BMAL1 led to reduced GPX4 degradation, while knockdown of BMAL1 promoted GPX4 degradation. In rescue experiments, knockdown of both BMAL1 and HMGB1 reversed the degradation of GPX4. In summary, we discovered that BMAL1 regulated ferroptosis in AML cells through the HMGB1-GPX4 pathway. One limitation of our study is that we did not investigate other potential pathways by which BMAL1 regulates ferroptosis in AML cells. Therefore, in future studies, it will be necessary to explore other potential pathways through which BMAL1 will regulate ferroptosis in AML cells.

Numerous studies have demonstrated that BMAL1 represents a viable target for the development of anti-cancer therapeutics (Wang et al. 2023a, b). One study has demonstrated an association between BMAL1 and resistance to bevacizumab in colorectal cancer (Burgermeister et al. 2019). Furthermore, our research findings indicated that BMAL1 was associated with AML cell proliferation and adverse prognosis. Therefore, it was hypothesized that BMAL1 might be implicated in the chemoresistance of AML cells. After conducting experiments on the impact of BMAL1 on the sensitivity of certain frrst-line cancer treatment drugs, we observed that knocking down BMAL1 increased the sensitivity of AML cells to the drugs venetoclax, dasatinib, and sorafenib. BCL-2 is highly expressed in hematologic malignancies, conferring anti-apoptotic properties to the tumors. Venetoclax is the first BCL-2 inhibitor that promotes apoptosis (Dhakal et al. 2022; Diepstraten et al. 2022; Sullivan et al. 2022). The combination of venetoclax with other drugs, such as azacitidine and decitabine, has been shown to significantly improve the rate of complete remission and patient survival. However, when used as a monotherapy, its efficacy is limited (Milnerowicz et al. 2023). In addition, a significant proportion of patients exhibit primary or acquired resistance to venetoclax (Dhakal et al. 2023). Current research indicates that the main mechanisms of resistance to venetoclax involve elevated expression of other apoptotic proteins (MCL-1, BCL-XL) and activation of alternative anti-apoptotic pathways. Consequently, the current focus of research is on overcoming resistance to venetoclax. Our research findings indicated that the inhibition of BMAL1 increased the sensitivity of AML cells to venetoclax. Targeting BMAL1 as a potential way to enhance the efficacy of venetoclax and may provide a breakthrough solution to addressing venetoclax resistance. However, further in-depth research is needed to explore the precise mechanisms underlying these results. Dasatinib is a novel and effective multi-target inhibitor which targets Abl, Src, and c-Kit (Keating 2017). *KIT* mutations are unfavorable prognostic factors in AML, indicating a higher relapse rate. The incidence of *KIT* mutations in AML is relatively high, approximately 60% (Al-Kali et al. 2023). Hence, the utilization of KIT inhibitors as a treatment strategy for AML patients harboring KIT mutations or exhibiting high KIT expression is highly viable. Presently, there are several ongoing clinical research endeavors dedicated to investigating the efficacy of this approach. The findings of this study provide valuable insights for future investigations on the combination therapy involving dasatinib. FLT3-ITD mutation is present in approximately 20% of AML patients (Burchert 2021). These patients have a higher risk of relapse and mortality, with a poor prognosis (Schnittger et al. 2002; Thiede et al. 2002; Brunet et al. 2012; Schlenk et al. 2014). As such, targeting FLT3 is an exceptionally effective approach for the treatment of AML. As a first-generation multi-targeted small molecule inhibitor of FLT3, sorafenib targets Raf kinase, VEGFR-2, c-KIT, and FLT3 (Wilhelm et al. 2006). It has demonstrated significant efficacy in suppressing the growth activity of AML cells carrying FLT3-ITD mutation. The current clinical evidence suggests that sorafenib administration can provide benefits in AML patients harboring FLT3-ITD mutation (Röllig et al. 2015; Deol et al. 2016; Battipaglia et al. 2017). Nevertheless, the incidence of adverse reactions is considerably higher during sorafenib therapy, thereby imposing limitations on its utilization (Zhang et al. 2008; Zauli et al. 2012; Roskoski 2020). Our study has revealed that reducing BMAL1 expression increases the responsiveness of AML cells to the anti-tumor medications venetoclax, dasatinib, and sorafenib. Our results offer valuable insights into the potential correlation between BMAL1 and drug resistance in tumors. Nevertheless, the precise mechanism underlying BMAL1's regulation of drug resistance in human tumors, including AML, remains elusive and has not been extensively investigated. The development of small molecule inhibitors that target BMAL1 in future research on AML treatment is a promising avenue that could potentially revolutionize AML therapy.

In summary, this study demonstrates the high expression of BMAL1 in AML and its potential association with an unfavorable prognosis. Furthermore, we have observed a correlation between BMAL1 and AML cell proliferation, cell cycle regulation, and chemotherapy resistance. Targeting BMAL1 may enhance the anticancer activity of dasatinib,venetoclax,and sorafenib for AML. Significantly, we have provided the first elucidation of the regulatory role of BMAL1 in ferroptoisis of AML cells through the HMGB1-GPX4 pathway. Given that BMAL1 is a potential drug target for intervention in AML treatment, further research on discovering potent and low-toxic BMAL1 inhibitors, as well as understanding the mechanism of BMAL1 in AML, is necessary.

### Supplementary Information

Below is the link to the electronic supplementary material.

Supplementary file1 (TIF 12330 KB)

Supplementary file2 (TIF 13685 KB)

Supplementary file3 (TIF 3564 KB)

Supplementary file4 (TIF 18721 KB)

Supplementary file5 (TIF 9216 KB)

Supplementary file6 (TIF 1564 KB)

Supplementary file7 (DOCX 23 KB)

Supplementary file8 (DOCX 22 KB)

## Data Availability

All experimental and analytical results of this study are included in the manuscript.
