# Supplementary figures and images for: Targeting BMAL1 reverses drug resistance of acute myeloid leukemia cells and promotes ferroptosis through HMGB1-GPX4 signaling pathway
Source: J Cancer Res Clin Oncol. 2024 May 4;150(5):231. doi: 10.1007/s00432-024-05753-y (PMC11069489; doi:10.1007/s00432-024-05753-y)

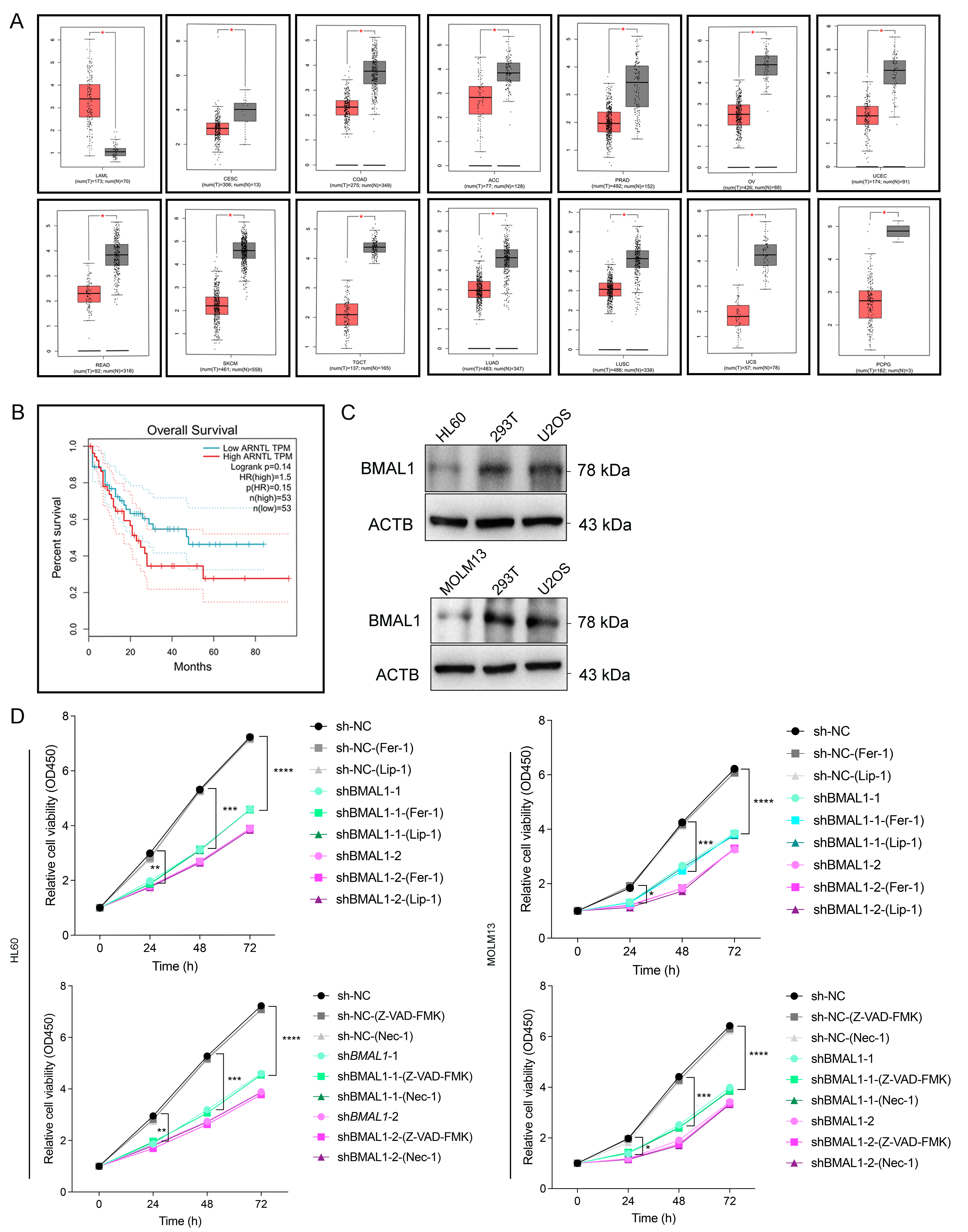

Supplement: Supplementary file 1 — Supplementary file1 (TIF 12330 KB) [file 432_2024_5753_MOESM1_ESM.tif]

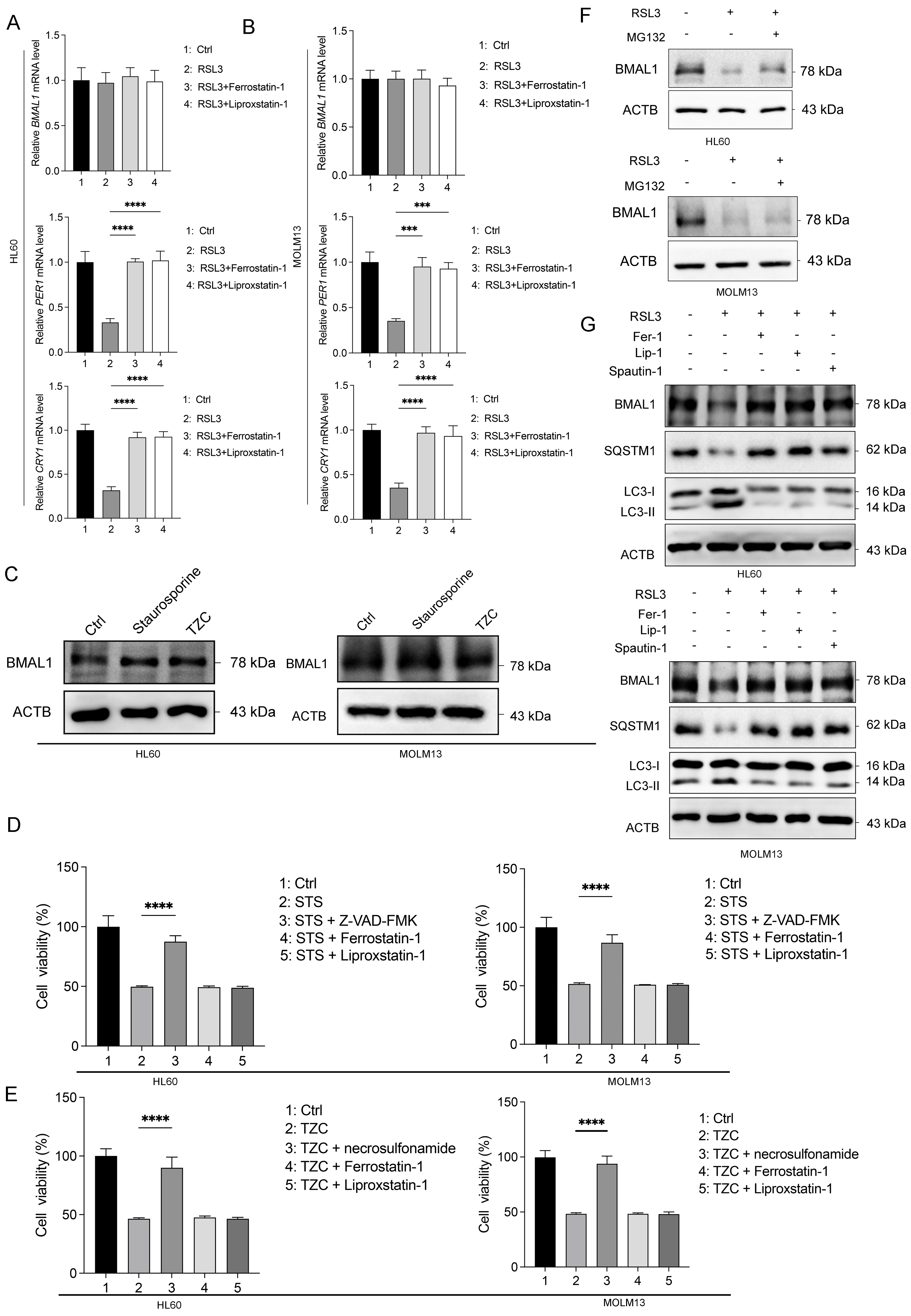

Supplement: Supplementary file 2 — Supplementary file2 (TIF 13685 KB) [file 432_2024_5753_MOESM2_ESM.tif]

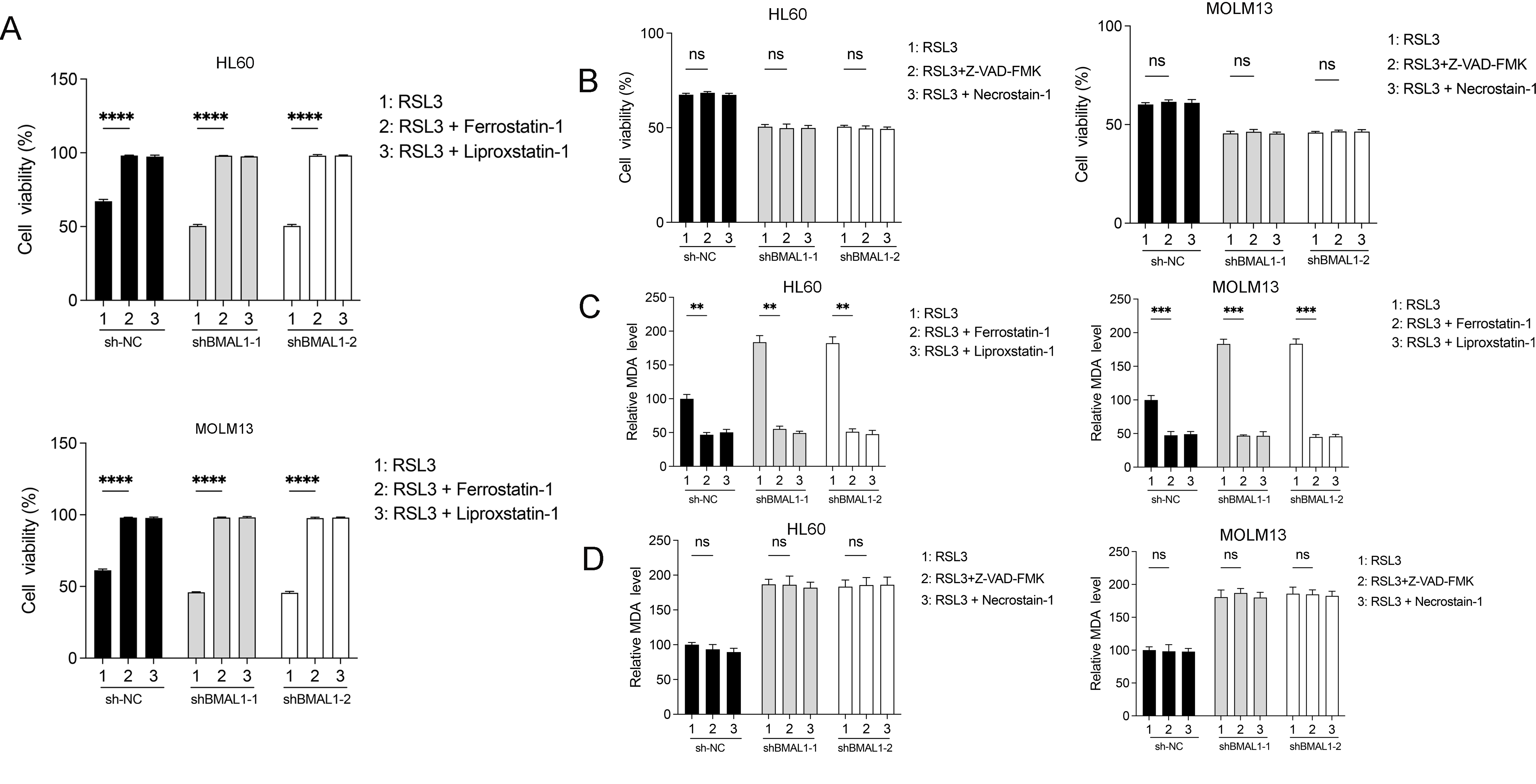

Supplement: Supplementary file 3 — Supplementary file3 (TIF 3564 KB) [file 432_2024_5753_MOESM3_ESM.tif]

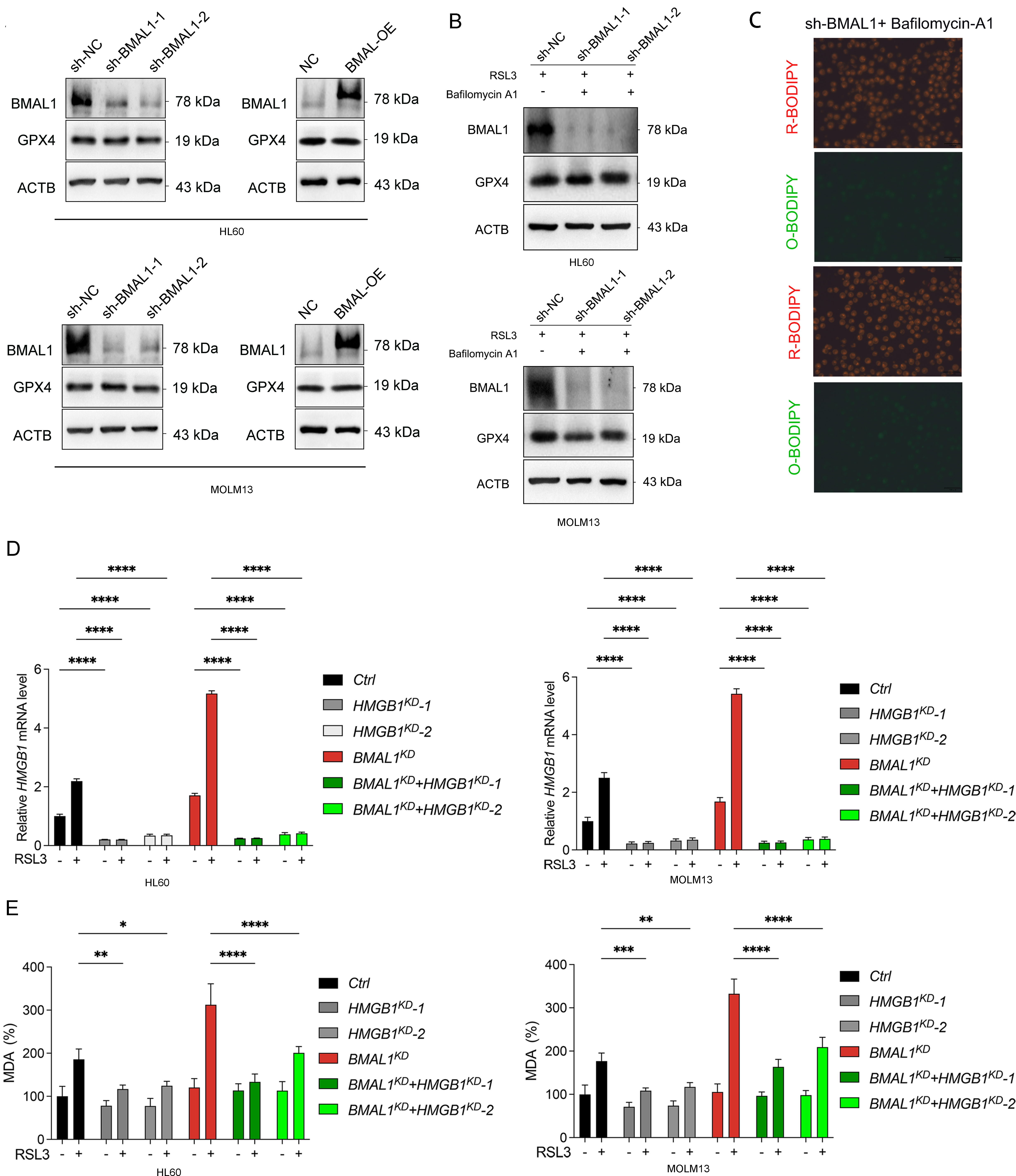

Supplement: Supplementary file 4 — Supplementary file4 (TIF 18721 KB) [file 432_2024_5753_MOESM4_ESM.tif]

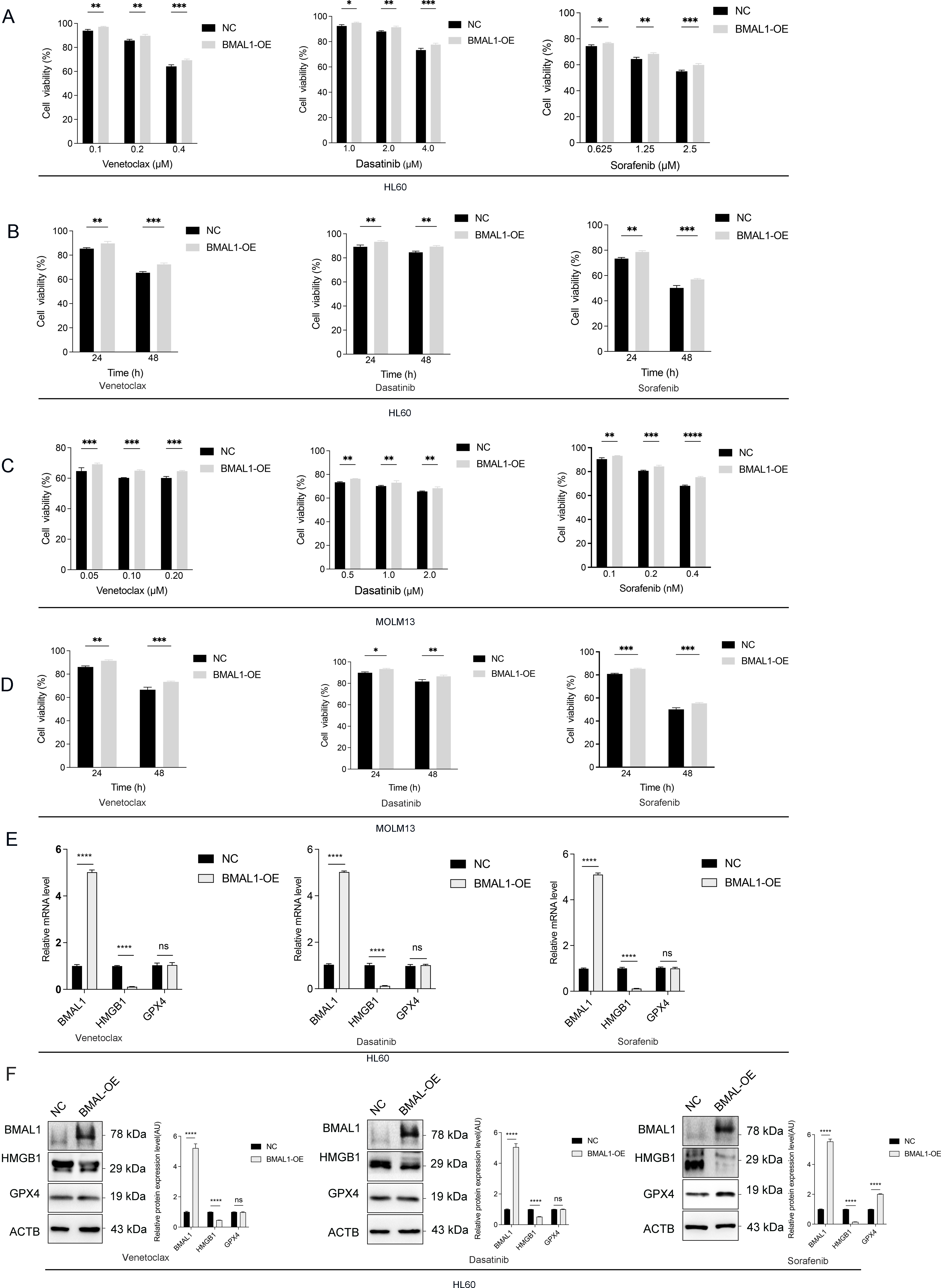

Supplement: Supplementary file 5 — Supplementary file5 (TIF 9216 KB) [file 432_2024_5753_MOESM5_ESM.tif]

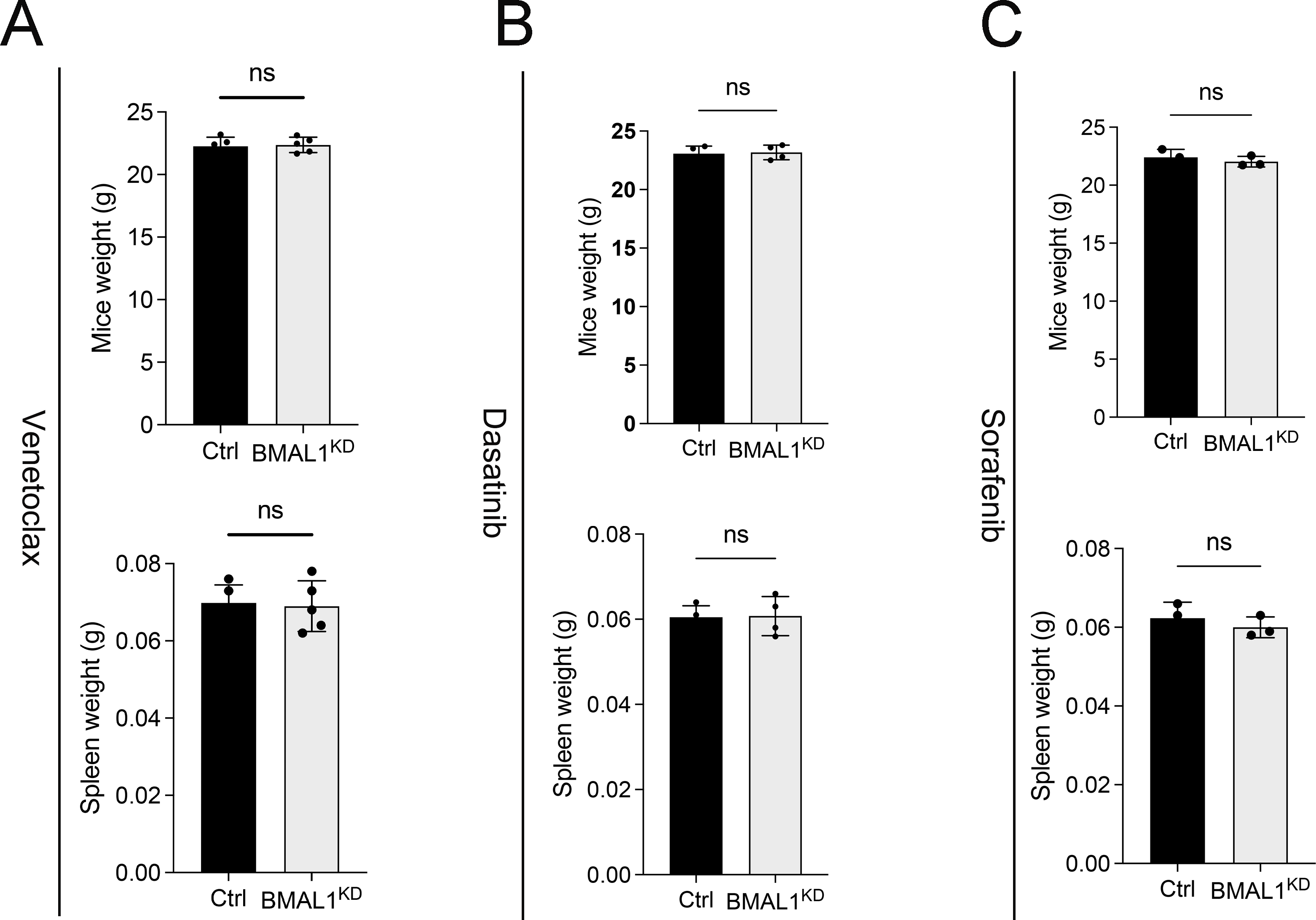

Supplement: Supplementary file 6 — Supplementary file6 (TIF 1564 KB) [file 432_2024_5753_MOESM6_ESM.tif]
