# Supplementary material for: Targeting BMAL1 reverses drug resistance of acute myeloid leukemia cells and promotes ferroptosis through HMGB1-GPX4 signaling pathway
Source: J Cancer Res Clin Oncol. 2024 May 4;150(5):231. doi: 10.1007/s00432-024-05753-y (PMC11069489; doi:10.1007/s00432-024-05753-y)
Supplement: Supplementary file 7 — Supplementary file7 (DOCX 23 KB) [file 432_2024_5753_MOESM7_ESM.docx]

**Targeting BMAL1 reverses drug resistance of acute myeloid leukemia cells and promotes ferroptosis through HMGB1-GPX4 signaling pathway**

Hong Zheng^1^, Ting Wu^3^, Zhi Lin^2,4,5^, Dan Wang^2,4,5^, Jing Zhang^3^, Ting Zeng^1^, Leping Liu^2,4,5^ , Jie Shen^2,4,5^,Mingyi Zhao^2,4,5^, Jia-Da Li^3*^, Minghua Yang^2,4,5*^

^1^Department of Pediatrics, The Xiangya Hospital, Central South University, Changsha, Hunan 410008, China

^2^Department of Pediatrics, The Third Xiangya Hospital, Central South University, Changsha, Hunan 410013, China

^3^Center for Medical Genetics, School of Life Sciences, Central South University, Changsha, Hunan 410013, China

^4^Hunan Clinical Research Center of Pediatric Cancer, The Third Xiangya Hospital, Central South University, Changsha, Hunan 410013, China

^5^ MOE Key Lab of Rare Pediatric Diseases, The Third Xiangya Hospital, Central South University, Changsha, Hunan 410013, China

** Corresponding author at:

1. Department of Pediatrics, The Third Xiangya Hospital, Central South University, Changsha, Hunan 410013, China. (M. Yang) ORCID: 0000-0003-3746-1209
2. Center for Medical Genetics, School of Life Sciences, Central South University, Changsha, Hunan 410013, China. (M. Li)

E-mail addresses: yangminghua@csu.edu.cn (M. Yang). [lijiada@sklmg.edu.cn](mailto:lijiada@sklmg.edu.cn) (M. Li).

**Supplementary figure 1** BMAL1 functions as a positive modulator in AML cells **A** Bioinformatics analysis from TGCA database was applied for protein expression of BMAL1 in tumor and normal tissues differential significantly. **B** Kaplan–Meier overall survival analyses was applied according to BMAL1 expression in AML patients. **C** Western blot analysis of the indicated proteins in HL60, MOLM13, 293T and U2SO cells. **D** Viability of controls and BMAL1-depleted HL60 and MOLM13 cells in the absence or presence of ferrostatin-1 (0.5 μM), liproxstatin-1 (0.5 μM), Z-VAD-FMK (20 μM) and Necrostain-1(30 μM) at the indicated time. Data represents the mean ± SD from three independent experiments; n.s. (no significance). Statistical significance in D was calculated by two-way ANOVA with Dunnett’s multiple comparison test. *p <0.05; **p <0.01; ***p <0.001. ****p < 0.0001.

**Supplementary figure 2** BMAL1 as an inhibitor of ferroptosis in AML cells. **A-B** Quantitative polymerase chain reaction (qPCR) analysis of the indicated mRNAs in HL60 and MOLM13 cells following treatment with RSL3 (0.5 μM) in the absence or presence of ferrostatin-1 (0.5 μM) or liproxstatin-1(0.5 μM) for 24 hours. **C** Western blot analysis of the indicated proteins in HL60 and MOLM13 cells following treatment with staurosporine (1 μM) or TZC [TNF (50 nM), Z-VAD-FMK (20 μM), and cycloheximide (10 μg/ml)] for 24 hours. **D** Viability of HL60 and MOLM13 cells following treatment with staurosporine (1 μM) for 24 hours in the absence or presence of Z-VAD-FMK (20 μM), ferrostatin-1 (0.5 μM), or liproxstatin-1 (0.5 μM). **E** Viability of HL60 and MOLM13 cells after treatment with TZC [TNF (50 nM), Z-VAD-FMK (20 μM), and cycloheximide (10 μg/ml)] for 24 hours in the absence or presence of necrosulfonamide (1 μM), ferrostatin-1 (0.5 μM), or liproxstatin-1 (0.5 μM). **F** Western blot analysis of the indicated proteins in HL60 and MOLM13 cells following treatment with RSL3 (1.0 μM) in the absence or presence of MG-132 (2.5 μM) for 24 hours. **G** Western blot analysis of the indicated proteins in HL60 and MOLM13 cells following treatment with RSL3 (1.0 μM) in the absence or presence of ferrostatin-1 (0.5 μM), liproxstatin-1 (0.5 μM) or spautin-1 (5 μM) for 24 hours. Data represents the mean ± SD from three independent experiments; n.s. (no significance). Statistical significance in (A-B), (D-E) was calculated by one-way ANOVA with Dunnett’s multiple comparison test. *p <0.05; **p <0.01; ***p <0.001. ****p < 0.0001.

**Supplementary figure 3** BMAL1 as an inhibitor of ferroptosis in AML cells. **A** Cell viability of both control and BMAL1-depleted HL60 and MOLM13 following treatment with RSL3(1.0 μM), ferrostatin-1 (0.5 μM) and liproxstatin-1 (0.5 μM). **B** Cell viability of both control and BMAL1-depleted HL60 and MOLM13 following treatment with RSL3(1.0 μM), Z-VAD-FMK (20 μM) and Necrostain-1(30 μM). **C** Analysis of MDA levels in both controls and BMAL1-depleted HL60 and MOLM13 cells following treatment with RSL3(1.0 μM), ferrostatin-1 (0.5 μM) and liproxstatin-1 (0.5 μM). **D** Analysis of MDA levels in both controls and BMAL1-depleted HL60 and MOLM13 cells following treatment with RSL3(1.0 μM), Z-VAD-FMK (20 μM) and Necrostain-1(30 μM). Data represents the mean ± SD from three independent experiments; n.s. (no significance). Statistical significance in (A-D) was calculated by two-way ANOVA with Dunnett’s multiple comparison test. *p <0.05; **p <0.01; ***p <0.001. ****p < 0.0001.

**Supplementary figure 4** BMAL1 regulates ferroptosis via HMGB1-GPX4 pathway.

**A** Western blot analysis of the indicated proteins in both controls, BMAL1-depleted and BMAL1-overexpression HL60 and MOLM13 cells. **B** Western blot analysis of the indicated proteins in both controls, BMAL1-depleted HL60 and MOLM13 cells following treatment with RSL3(1.0 μM) in the absence or presence of bafilomycin A1 (50.0 nM) for 24 hours. **C** Analysis of lipid peroxidation in the indicated gene knockdown HL60 and MOLM13 cells after treatment with RSL3 (1.0 μM) in the absence or presence of bafilomycin A1 (50.0 nM) for 24 hours. **D** Quantitative polymerase chain reaction (qPCR) analysis of the *HMGB1* mRNAs in the indicated gene knockdown HL60 and MOLM13 cells after treatment with RSL3 (1.0 μM) for 24hour. **E** Analysis of MDA levels in the indicated gene knockdown HL60 and MOLM13 cells after treatment with RSL3 (1.0 μM) for 24 hours. Data represents the mean ± SD from three independent experiments; n.s. (no significance). Statistical significance in (D-E) was calculated by two-way ANOVA with Dunnett’s multiple comparison test. *p <0.05; **p <0.01; ***p <0.001. ****p < 0.0001.

**Supplementary figure 5** Targeting BMAL1 sensitizes AML cells to targeted agents. **A** Cell viability of both control and BMAL1-overexpressing HL60 cells treated with venetoclax, dasatinib and sorafenib at different concentrations as indicated for 24 hours. **B** Cell viability of both control and BMAL1-overexpressing HL60 cells treated with venetoclax (0.2 μM), dasatinib (2.0 μM) and sorafenib (0.3 μM) at different time points as indicated. **C** Cell viability of both control and BMAL1-overexpressing MOLM13 cells treated with venetoclax, dasatinib and sorafenib at different concentrations as indicated for 24 hours. **D** Cell viability of both control and BMAL1-overexpressing MOLM13 cells treated with venetoclax (0.02 μM), dasatinib (0.1 μM) and sorafenib (0.2 nM) at different time points as indicated.

**E** Quantitative polymerase chain reaction (qPCR) analysis of the *HMGB1* and *GPX4* mRNAs in both controls, BMAL1-overexpressing HL60 cells after treatment with venetoclax (0.4 μM), dasatinib (4.0 μM) and sorafenib (0.625 μM) for 24 hours. **F** Western blot analysis of the indicated proteins in both controls, BMAL1-overexpressing HL60 cells after treatment with venetoclax (0.4 μM), dasatinib (4.0 μM) and sorafenib (0.625 μM) for 24 hours. Data represents the mean ± SD from three independent experiments; n.s. (no significance). Statistical significance in (A-E) was calculated by two-way ANOVA with Dunnett’s multiple comparison test. *p <0.05; **p <0.01; ***p <0.001. ****p < 0.0001.

**Supplementary figure 6** BMAL1 regulates ferroptosis and anticancer agents’ efficacy in *vivo*. **A-C** The weight of indicated mice and spleen following treatment with venetoclax, sorafenib, dasatinib. Statistical significance in (A-D) was calculated by wo-tailed unpaired t test. *p <0.05; **p <0.01; ***p <0.001. ****p < 0.0001.
