# Supplementary material for: Targeting BMAL1 reverses drug resistance of acute myeloid leukemia cells and promotes ferroptosis through HMGB1-GPX4 signaling pathway
Source: J Cancer Res Clin Oncol. 2024 May 4;150(5):231. doi: 10.1007/s00432-024-05753-y (PMC11069489; doi:10.1007/s00432-024-05753-y)
Supplement: Supplementary file 8 — Supplementary file8 (DOCX 22 KB) [file 432_2024_5753_MOESM8_ESM.docx]

**Targeting BMAL1 reverses drug resistance of acute myeloid leukemia cells and promotes ferroptosis through HMGB1-GPX4 signaling pathway**

Hong Zheng^1^, Ting Wu^3^, Zhi Lin^2,4,5^, Dan Wang^2,4,5^, Jing Zhang^3^,Ting Zeng^1^, Leping Liu^2,4,5^ , Jie Shen^2,4,5^,Mingyi Zhao^2,4,5^, Jia-Da Li^3*^, Minghua Yang^2,4,5*^

^1^Department of Pediatrics, The Xiangya Hospital, Central South University, Changsha, Hunan 410008, China

^2^Department of Pediatrics, The Third Xiangya Hospital, Central South University, Changsha, Hunan 410013, China

^3^Center for Medical Genetics, School of Life Sciences, Central South University, Changsha, Hunan 410013, China

^4^Hunan Clinical Research Center of Pediatric Cancer, The Third Xiangya Hospital, Central South University, Changsha, Hunan 410013, China

^5^ MOE Key Lab of Rare Pediatric Diseases, The Third Xiangya Hospital, Central South University, Changsha, Hunan 410013, China

** Corresponding author at:

1. Department of Pediatrics, The Third Xiangya Hospital, Central South University, Changsha, Hunan 410013, China. (M. Yang) ORCID: 0000-0003-3746-1209
2. Center for Medical Genetics, School of Life Sciences, Central South University, Changsha, Hunan 410013, China. (M. Li)

E-mail addresses: yangminghua@csu.edu.cn (M. Yang). [lijiada@sklmg.edu.cn](mailto:lijiada@sklmg.edu.cn) (M. Li).

**Supplementary Table 1** Clinical characteristics of AML patients used in this study.

| NO.of patients | Age(yr) | Gender | AML type | Disease status | Mutational profile | Karyotype | MRD |
| --- | --- | --- | --- | --- | --- | --- | --- |
| 1 | 11 | female | M2 | primary | ETO/AML1、KIT、WT1 | t(8;21)(q22;q22) | MRD- |
| 2 | 12 | female | M2b | primary | ETO/AML1, KIT, RUNX1-RUNX1T1 | t(8;21)(q22;q22) | MRD- |
| 3 | 4 | female | M5b | primary | MLL/AF9 | normal | MRD- |
| 4 | 5 | male | M2a | primary | ETO/AML1, KIT, FLT3-ITD | normal | MRD- |
| 5 | 11 | male | M5a | primary | MLLAF10,NRAS,KRAS | chromosome aberration | MRD- |
| 6 | 12 | male | M2b | primary | ETO/AML1,FLT3-ITD,RUNX1-RUNX1T1,PIK3R3-IGSF1 | t(8;21)(q22;q22) | MRD：1.5% |
| 7 | 3 | female | M1 | primary | FLT3-ITD,NPM1-CCDC28A | normal | MRD：0.27% |
| 8 | 11 | male | M4 | primary | CBFB-MYH11,NRAS | normal | MRD- |
| 9 | 3 | male | M2 | primary | IKZF1-FIGNL1,FLT3-ITD,CEBPA | normal | MRD- |

**Supplementary Table 2** The upstream sequence of HMGB1 TSS (Transcription Start Site).

gagctcTTTCTGCGGAGGGATTACGCTGACGAAAGAGACCTGCTTGCGCGTCGCTGTTCCGTGGTCCGCGCGAGCGTGGTCGGGAGCCGCTGGTTCCTGGGGTGACCCGCGGAGGTGGGAGAGGGAAGGGCTTCCGAAGCCGGCGGGGGTGCCATGGACCCTCTCCGCCGGCGCGGCCTTCACAGCTGGGCCGCGCCGGGCATCCGTAGTCCGCTCTCCCAAAGCCTCGGTGGAGCTGAAGCTGCCACAGAGTGCATGTTCACAAAGGGTCATCACACACGGAGCTGCCCCTCCCTGTCTCCCTAGAGCCCATCTTCGAGGCCAGGGGCTTTTCTACCAGGATTCTGGGGTGTTTCTCCTCCTTTCCTCCCTCCCAGATCTTCTCACGGTAAGGGGAGCAGCGAAAGCGCAGGGACTTTGCATTCCACGACCCGTTCTGACTAGTCAACAGCCGATCTGTCCCTGCTGCTCTAATTCCAGCTGCCCTGCCTTGTTTTAACTTCAGAGAAAGGGGGAGTTCTCATTTGATAAGTTTAAGCCTTTGCTTTCGTAGGAAGGTCATGTGGCTTAAGGGACATCGTGACCGCGTATGCTATTTCTGCCTGTGCATTCTAAATCTTGGGGGCAGCATATTCCAGAAGTCCTTTTGGTCGATTGGTTCTGTGTCCTAGGATAACAATTTGTAGTTTCTGACCATTTCTTTACAGAAAAACCACAATTGGTATTTTGGACTGCGGGGTTTTTTAGTGGTCTCAAACTAAATGATTATATTCTGGAATAATGCTGACAATTTGGATAGGGTGGTGTGGAGGAAACAAGTCTCGTGTAGAAGAAATTATTTAGTAAAAAGGATTTTAGTTTTTGGTACTTCTGAATGCAAATGGCCAAGGAATCCAGCAGTTTGTTGGGGTTTCTCCAGACAAAAATAGGCTGGTTAGTTTGTTTACCTTTTTCTGTTGCTTTACTAGATTGTTAATGTATTTGTATATTTAGATCTAGCTAGTTAGGTTTTTCTTTTTTATGAATTGTGATTAAAATTGGAGAAAGTAGTACTTCCTCCTGAAATAGACAAAACTCAGAGGGTTGTTTTCTTGTTCACCACTGCTACTTCCATTTTTAGAATGGAATACACTATTTGTAAAAAACAAACTTGCTGAGTATTAACTCATAAATTGAACAAAACAATTGGTTCAGTTAAAATGTAGCTAAAATTGTAAGACTGCAAAACAACATCACCAAACTGAATTGTATTTTTTAAAACGTTGTCTGCAAAGTTGAATTTTAGTCTTTCAGTGTAAATTTGGTATTTTTTCAGAAGAAAAATTAGTAACAGAAACAAAATGGAGGCGGGGCTTGAGTTGTAAAGAGAAAGGAAGGGATTTTAGTGTGTTCTGTGTTTTCGCTCTAGAGCTTTTTCCTCTTGTAAAAGGTAGTACAAGACTTATAAAGGGAAGAACTTTGGTACAGGAGATCAGCAGAGATTGACTAGAAAACCCCTTTCGTTTATATTATAGAGTAACGGTCCCAAGTTGAGAATACTGTATACATTTCTGGTTATTCCTATATATTTGAAATGCTTTTTTCTACATATCCTGGTCCCAGAGAGAGTGCAGTACCTTCCAGTGGGATTAAAGCTAATTGCGTCCATCGCTTGTCAGCAGTGTGGGACAGAAATTTTATTGACCTATTTCCAGTTATAAACTCACATGATTTAAAGGGAGGAAGCTTTTATTAAATTAATAACTAACGAACAGCTGCAGTAGTTCAGTGCTTCTGTATGATTAGGATTTTCTCTTTTTTTCTCTCATTTTAGCCACTCACGATGTCAGCTCGTGCACCTTCTACTAAGTTAGATTCCTTAACCACAATACAAGTCTTTGGCCCAAACCTGGgctagc
